# The role of prenatal and perinatal factors in eating disorders: a systematic review

**DOI:** 10.1007/s00737-020-01057-5

**Published:** 2020-08-07

**Authors:** Enrica Marzola, Fabio Cavallo, Matteo Panero, Alain Porliod, Laura Amodeo, Giovanni Abbate-Daga

**Affiliations:** Department of Neuroscience “Rita Levi Montalcini”, University of Turin, via Cherasco 15, 10126 Turin, Italy

**Keywords:** Eating disorders, Anorexia nervosa, Bulimia nervosa, Pregnancy complications, Obstetric complications

## Abstract

Numerous studies showed that factors influencing fetal development and neonatal period could lead to lasting alterations in the brain of the offspring, in turn increasing the risk for eating disorders (EDs). This work aims to systematically and critically review the literature on the association of prenatal and perinatal factors with the onset of EDs in the offspring, updating previous findings and focusing on anorexia nervosa (AN) and bulimia nervosa (BN). A systematic literature search was performed on Pubmed, PsycINFO, and Scopus. The drafting of this systematic review was conducted following the PRISMA statement criteria and the methodological quality of each study was assessed by the MMAT 2018. A total of 37 studies were included in this review. The factors that showed a more robust association with AN were higher maternal age, preeclampsia and eclampsia, multiparity, hypoxic complications, prematurity, or being born preterm (< 32 weeks) and small for gestational age or lower birth size. BN was only associated with maternal stress during pregnancy. Many methodological flaws emerged in the considered studies, so further research is needed to clarify these inconsistencies. Altogether, data are suggestive of an association between prenatal and perinatal factors and the onset of EDs in the offspring. Nevertheless, given the methodological quality of the available literature, firm conclusions cannot be drawn and whether this vulnerability is specific to EDs or mental disorders remains to be defined. Also, a strong need for longitudinal and well-designed studies on this topic emerged.

**Electronic supplementary material:**

The online version of this article (10.1007/s00737-020-01057-5) contains supplementary material, which is available to authorized users.

## Introduction

Eating disorders (EDs) are complex mental illnesses characterized by unknown etiology, with many putative risk factors (Fairburn and Harrison 2003; Dalle Grave 2011; Jacobi et al. 2011) facilitating the ED onset. Genetic risk factors are of great importance, with genes impacting on the development of both anorexia nervosa (AN) and bulimia nervosa (BN) as well as on their predisposing traits (Trace et al. 2013; Baker et al. 2017). An altered neurodevelopment has been implicated in the pathogenesis of several mental disorders (Katzman et al. 1997; Chowdhury et al. 2003; King et al. 2018) such as schizophrenia (Geddes and Lawrie 1995; Verdoux et al. 1997; Geddes et al. 1999; Cannon et al. 2000), attention deficit hyperactivity disorder (Lindström et al. 2006), and autism (Gardener et al. 2009). With more detail, hypoxic complications and prematurity have been associated with schizophrenia risk (Geddes and Lawrie 1995; Verdoux et al. 1997; Geddes et al. 1999; Cannon et al. 2000), leading to the formulation of a “neurodevelopmental hypothesis for schizophrenia” (Rapoport et al. 2012).

Several studies (Gillberg et al. 1994; Connan et al. 2003) linked prenatal and perinatal complications (Raevuori et al. 2014) to EDs, with the hypothesis that subtle neurological damages (Gillberg et al. 1994), neuropsychological disabilities (Galderisi et al. 2003), and nonreversible morphological brain changes could ease the disorder onset. Krug and coworkers (Krug et al. 2013) systematically reviewed the literature on obstetric complications (OCs) and EDs, selecting 14 articles, and performing a meta-analysis where possible. Conflicting results emerged, so their meta-analysis found a nonsignificant association between instrumental delivery and prematurity and ED risk. More recently, a descriptive review of 22 articles focusing on additional risk factors (e.g., the role of sex hormones, maternal status, and maternal EDs) found once more mixed results (Raevuori et al. 2014). Another review of 13 studies (Jones et al. 2017) focused instead on “fetal programming” as a model on how stimuli/insults occurring during critical or sensitive periods of fetal development could have physiological effects that unfold across the life span. The latter review introduced new risk factors, such as maternal stress during pregnancy, but finding again controversial results. Therefore, the aim of this systematic review is twofold: (a) to critically review the updated literature on the association of prenatal and perinatal factors with the onset of EDs in the offspring and (b) to expand knowledge on the critical points that need to be addressed by future lines of research.

## Methods

### Search strategy and selection criteria

The drafting of this systematic review was conducted following the PRISMA statement criteria (Moher et al. 2009), and the studies included in this systematic review have been evaluated by the MMAT (Mixed Methods Appraisal Tool, 2018 version; Hong et al. 2018).

A systematic literature search was done between March 1 and May 1, 2019, including online database searches, namely, Pubmed, PsycINFO, and Scopus, and journal hand searching to ensure a wide inclusion of eligible studies.

The search was designed to include those studies published since 1998; this is a reasoned choice as the purpose of this systematic review was to analyze the most recent studies on the topic. All full-text studies published were included if they met the following inclusion criteria:

- Study type criteria: cohort study and case-control study
- Eating disorder assessment criteria (EDs): DSM (III, III-R, IV, IV-TR, 5) diagnosis, ICD (8,9,10) diagnosis, clinical diagnosis or self-report diagnosis, or ED symptomatology obtained with questionnaire scores
- Risk assessment criteria: obstetric birth records, parents recall, and/or a combination of both

The following list of terms were included and combined together in different search lines: “eating disorders,” “anorexia nervosa,” “bulimia nervosa,” “pregnancy complications,” “obstetric complications,” “prenatal risk factors,” “perinatal risk factors,” “gestational diabetes,” “hypertensive disease in pregnancy,” “maternal anemia,” “preeclampsia,” “eclampsia,” “maternal viral infection,” “season of birth,” “vit. D levels during pregnancy,” “prenatal sex hormones,” “dysmaturity,” “preterm birth,” “mother weight,” “mother smoke habit,” “maternal stress,” “maternal anxiety,” “placenta previa,” “pregnancy bleeding,” “breech delivery,” “induced labor,” “inertia uteri,” “premature rupture of the membrane,” “forceps,” “cesarean section,” “vaginal instrumental delivery,” “vacuum extraction,” “cephalohematoma,” “umbilical cord wrapped around the neck,” “placental infarction,” “cyanosis,” “jaundice,” “need for resuscitation,” “need for oxygen,” “need for intubation,” “birth weight,” “prematurity,” “tremors,” “hypothermia,” “hypotonia,” and “neuromuscular disturbances*.*”

**Figure 1:**
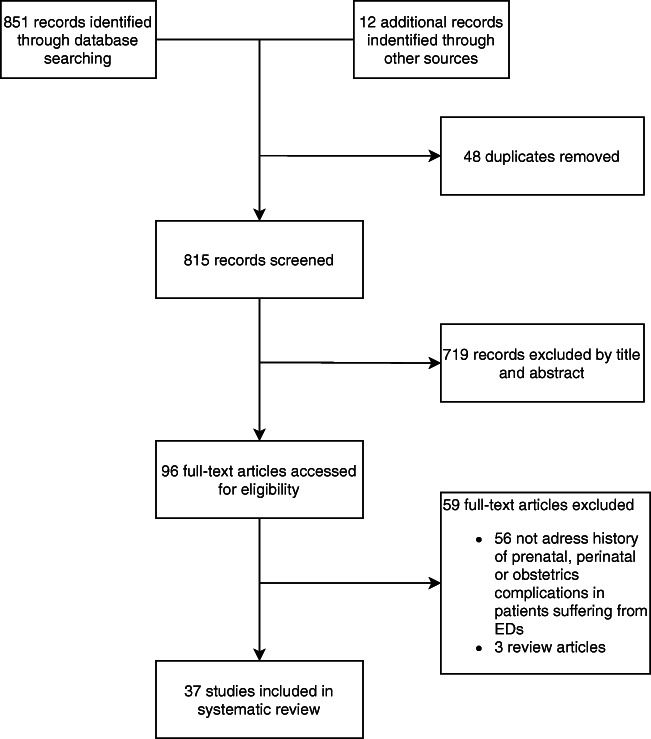
Study selection process

Two investigators (E.M., F.C.) screened the title and abstracts of 851 studies identified through the search using the inclusion criteria. Full-text articles were retrieved for all studies that met the inclusion criteria or required more information than was provided in the abstract. The reasons for excluding the studies were documented and shown in Fig. 1.

### Data analysis

All database search results were imported into Zotero. Duplicate entries were removed before the screening. Data from the studies were extracted and summarized (see Table 1 and Supplementary Materials). Data extracted included authors, year, study type, sample size, psychiatric assessment, maternal factors, pregnancy complications, obstetric complications, neonatal factors, and main findings.

**Table 1:** Article summary of study results included in this review

Assessed conditions								
Authors	Study type	Sample	Maternal factors	Pregnancy complications	Obstetric complications	Neonatal factors	Main findings	Quality rating
Cnattingius et al. 1999	Case-control study with randomly selected control (Swedish Inpatient Register—1973–1984)	AN (*n* = 781), HCs (*n* = 3905)	Maternal age	Hypertensive disease, diabetes, bleeding during pregnancy, multiparity	Inertia uteri, forceps, or vacuum delivery, preterm rupture of the membranes, cesarean section, cephalohematoma, other trauma	Gestational age, birth weight and birth weight for gestational age, Apgar score, jaundice	AN diagnosis was associated with maternal age, very preterm birth (< or = 32 weeks), small for gestational age in very preterm birth, cephalohematoma	5
Morgan et al. 2000	Case-control study	BN (*n* = 935), patients with AN history and BN diagnosis (*n* = 227) general population data	–	–	–	Month of birth	BN diagnosis is not associated with a specific month of birth AN history and BN diagnosis are associated with a peak season of birth in March	4
Shoebridge & Gowers et al. 2000	Case-control study	AN (*n* = 40) and HCs (*n* = 40)	–	Antepartum hemorrhage	Toxemia, previous obstetric complications, forceps delivery, cesarean section	Mean birth weight, gestation length (full-term, 36 weeks, 32 weeks) APGAR score, the baby looked after in a special care baby unit	No association was found	2
Foley et al. 2001	Twin cohort study (Virginia Twin Registry, *n* = 2352)	AN (*n* = 7), broadly defined AN (bdAN) (*n* = 71), BN (*n* = 42), broadly defined BN (bdBN) (*n* = 100), twin control (*n* = 1586)	–	High blood pressure, vaginal bleeding, seizures or toxemia, German measles, any other complication, any prenatal complication	Premature contractions, labor lasting more than 24 h, breech delivery, cesarean delivery, forceps delivery, cord wrapped around the neck, blue at birth, required an incubator, any perinatal complication	Birth weight, gestational age, jaundice, failure to breathe at first, convulsions, blood transfusion	AN: low gestational age, # prenatal maternal complications bdAN: prenatal maternal complications BN: # prenatal maternal complications bdBN: # prenatal maternal complications	3
Feingold et al. 2002	Historical prospective cohort (Thomas Jefferson University Hospital 1979–1981, *n* = 86)	Infants born preterm (*n* = 84)	Mother older than 40 years, alcohol abuse	Preeclampsia, eclampsia, chorioamnionitis, urinary tract infection, cervicitis, asthma, hypertension, hyperemesis gravidarum, cervical cancer, vaginal bleeding, multiparity	Premature rupture of the membranes, cesarean delivery, breech or transverse delivery, abruptio placentae, placenta previa, incompetent cervix, the total number of complications	Small for gestational age	No association with ED symptomatology for any of the studied variables	4
Lindberg and Hjern 2003	Population cohort study (Swedish Register Data 1973–1982 *n* = 989,871)	AN (*n* = 1122), HCs (*n* = 988,749)	Maternal age	Preeclampsia	Premature rupture of the membranes, placental abruption, breech delivery, cephalohematoma, neonatal O2 necessity	Gestational age, size for gestational age, prematurity, Apgar score, distress	AN diagnosis was associated with maternal age (> 25 years), gestational age at birth (23–32 weeks), preeclampsia, premature rupture of the membranes, low Apgar, O_2_ necessity, and distress, cephalohematoma, breech delivery	4
Montgomery et al. 2005	Cohort study (British cohort study 1970 *n* = 4046)	BN (*n* = 100), HCs (3946)	Maternal age at delivery, smoke habit, occupation, social class, psychiatric morbidity, BMI	–	–	–	Self-reported diagnosis of BN is associated with maternal smoke during pregnancy	3
Favaro et al. 2006	Birth cohort study (Padua birth cohort 1971–1979)	AN (*n* = 114), BN (*n* = 73), HCs (*n* = 554)	Maternal age, social class	Bleeding, preeclampsia, maternal diabetes, threatened miscarriages, and anemia	Inertia uteri, breech delivery, premature rupture of the membranes, breech delivery, placental infarction or abruption, placenta previa, meconium staining of the amniotic fluid, forceps or vacuum extraction, the umbilical cord wrapped around the infant’s neck, cephalopelvic need for resuscitation O_2_, and intubation, disproportion, # of obstetric complications, cephalohematoma	Small for gestation age, birth weight, cyanosis, respiratory and cardiac problems, jaundice, neuromuscular disturbances such as hyporeactivity, hypotonia, and tremors, hypothermia	AN diagnosis was associated with diabetes, anemia, preeclampsia, placental infarction, Ponderal Index < 25, neonatal cardiac problems, neonatal hyporeactivity, # of complications, the umbilical cord wrapped around the neck BN was associated with neonatal hyporeactivity, early feeding difficulties, placental infarction, low birth weight for gestational age, # of complications	5
Klump et al. 2006	Twin cohort study (Michigan State Twin Study)	FSS (*n* = 113)	–	Levels of prenatal testosterone (indirectly studied by 2D:4D ratio)	–	–	ED symptomatology was associated with lower levels of prenatal testosterone (higher 2D:4D ratio)	2
Culbert et al. 2008	Twin cohort study (Michigan State Twin Study)	FSS (*n* = 304), FOS (*n* = 59), MOS (*n* = 54), MSS (*n* = 165), HCs (*n* = 69)	–	Levels of prenatal testosterone (indirectly studied by OS and SS twin study)	–	–	Highest levels of disordered eating were observed for fSS twins, followed by fOS twins, mOS twins, and mSS twins	3
Favaro et al. 2008	Birth cohort study (Padua birth cohort 1971–1979)	AN (*n* = 66), BN (*n* = 44), HCs (*n* = 257)	Maternal age	Vaginal bleeding, preeclampsia, maternal diabetes, threatened miscarriages, and anemia	Inertia uteri, breech delivery, premature rupture of the membranes, placental infarction or abruption, placenta previa, meconium staining of the amniotic fluid, forceps or vacuum extraction, the umbilical cord wrapped around the infant’s neck, cephalohematoma, need for resuscitation O_2_ and intubation	Small for gestational age, birth weight, cyanosis, respiratory and cardiac problems, jaundice, neuromuscular disturbances such as hyporeactivity, hypotonia, and tremors, hypothermia	Preterm birth and neonatal dysmaturity were associated with high harm avoidance in the offspring affected by EDs	4
Raevuori et al. 2008	Twin cohort study (FinnTwin 16, 1975–1979 sample *n* = 2426)	2426 female twins with known zigosity (OS dizygotic *n* = 793, SS dizygotic *n* = 765, monozygotic *n* = 868), 1,962 male twins (OS dizygotic *n* = 717, SS dizygotic *n* = 705, monozygotic *n* = 540)	–	Levels of prenatal testosterone (indirectly studied by OS and SS twin study)	–	–	Opposite-sex twin pairs were not significantly different from monozygotic or same-sex dizygotic twins (female) in the association with AN or BN	4
Baker et al. 2009	Swedish twin study of child and adolescent development (TCHAD) 1985–1986	*n *= 439 identical females, *n n* = 213 fraternal females, *n *= 461 identical males, *n* = 344 fraternal males, *n* = 371 opposite-gender twin pairs	–	Levels of prenatal testosterone (indirectly studied by twin study)	–	–	ED symptomatology was not associated with twin types	3
Nicholls and Viner 2009	Prospective birth cohort (British cohort study, *n* = 16,567)	AN (*n* = 101), HCs (*n* = 11,261)	Smoke habit, ethnicity	Gestational diabetes, maternal anemia	Perinatal hypoxia	Birth weight, gestational age, prematurity (< 37 weeks)	No significant differences between AN and HCs	3
Smith et al. 2010	Cross-sectional observational study	Male college students (*n* = 204)	–	Levels of prenatal testosterone (indirectly studied by 2D:4D ratio)	–	–	Higher testosterone exposure (lower 2D:4D ratio) was associated with less ED symptoms, greater drive for muscularity, diminished drive for leanness	2
Wehkalampi et al. 2010	Case-control study (Helsinki Study of Very Low Birth Weight Adults 1978–1985 (*n* = 255)	Very low birth weight adults (*n* = 255), term (*n* = 189)	Maternal age, smoke habit, BMI, education	–	–	Gestational age, very preterm birth (< 32 weeks), very low birth weight—VLBW (< 1500 g)	In both sexes, EDI-2 scores were lower in VLBW individuals than in HCs	3
Coombs et al. 2011	Observational study on a Midlands High School (UK) students	*n* = 132 pupils (age 11–14)	–	Levels of prenatal testosterone (indirectly studied by 2D:4D ratio)	–	–	No strong association was found	2
Favaro et al. 2011	Birth cohort study (Padua Birth Cohort 1970–1984, *n* = 27,682)	AN (*n* = 402), HCs (*n* = 26,950)	Maternal education, maternal, and social class	Maternal exposure during pregnancy to chickenpox, measles, rubella, or influenza	–	Month of birth	AN diagnosis was associated with the exposure to rubella and chickenpox at the 6th of pregnancy. Being born in June was associated with AN	4
Quinton et al. 2011	Case-control study	AN (*n* = 25), BN (*n* = 26), HCs (*n* = 99)	–	Levels of prenatal testosterone (indirectly studied by 2D:4D ratio)	–	–	AN was associated with low 2D:4D ratio (higher prenatal testosterone) BN: high 2D:4D ratio was associated with lower prenatal testosterone	2
Nosarti et al. 2012	Historical population- based cohort study (Swedish Birth Register 1973–1985 and Hospital Discharge Register *n* = 1,301,522)	ED (*n* = 997), other psychiatric disorder and HCs	Maternal age, maternal education, maternal psychiatric family history	Parity	–	Gestational age, birth weight for gestational age, newborn sex, Apgar score at 5 min	ED: gestational week (<32 weeks)	4
Lydecker et al. 2013	Three twin cohort study (MATR (US), NIPHTP (Norway), STAGE (Sweden))	US: OS (*n* = 481), SS (*n* = 1022) Norway: OS (*n* = 345), SS (*n* = 1430) Sweden: OS (*n* = 2433), SS (*n* = 7000)	–	Levels of prenatal testosterone (indirectly studied by OS and SS twin study)	–	–	No association between co-twin sex and EDs	4
Allen et al. 2013	Population cohort (Western Australian Pregnancy Cohort (Raine) *n* = 2900)	ED (*n* = 98), HCs (*n* = 428)	Maternal age, BMI, drinking alcohol, smoking cigarettes, education, family income	Serum 25(OH)D level at 18 weeks, kidney disease or dysfunction, urinary tract infection, thyroid dysfunction	–	Gestational age at birth, season of birth, weight, preterm birth (< 37 weeks), newborn sex	EDs were associated with low quartile serum 25(OH)D level and season of birth (spring), kidney disease or dysfunction, sex (female) BN was associated with low quartile serum 25(OH)D level and season of birth (spring)	3
Culbert et al. 2013	Twin cohort study (Michigan State Twin Study)	FSS, fOS, mOS, mSS (*n* = 394), HCs (*n* = 63)	–	Levels of prenatal testosterone (indirectly studied by OS and SS twin study)	–	–	No differences were observed in the levels of disordered eating attitudes in opposite-sex and same-sex twins in pre-early puberty. During later phases of puberty, females from opposite-sex twin pairs exhibited lower disordered eating attitudes than females from same-sex twin pairs	3
Taborelli et al. 2013	Case-control study (Sister Pair Study)	AN (*n* = 94), BN (*n* = 63), HCs (*n* = 157)	Anxiety during pregnancy (questionnaire)			–	Anxiety during pregnancy was associated with AN, but not BN	4
Vellisca et al. 2013	Case-control study	AN (*n* = 210), general population data	–	–	–	Month of birth	AN diagnosis was not associated with the month of birth	4
Winje et al. 2013	Case-control study	AN (*n* = 4045), HCs with the same year of birth, sex, and region of birth	–	–	–	Month of birth	No significant differences between AN and HCs	4
Goodman et al. 2014	Population cohort study (Swedish Register Data, 1975–1998, *n* = 2,135,279)	AN (*n* = 7351), BN (*n* = 2804), other eating disorders (*n* = 10,408), HCs (*n* = 2015,862)	Maternal age, maternal education, number of full siblings, number of half-siblings, eating disorder in mother, multiple smoke habits	Multiparity	Premature rupture of the membranes, cesarean section, instrumental delivery, cephalohematoma, other birth trauma	Gestational age, birth weight for gestational age, birth length for gestational age, APGAR score	AN was associated with maternal age, multiparity, lower gestational age (dose-response). Weak evidence of cesarean and instrumental delivery and birth trauma other than cephalohematoma AN was negatively associated with higher maternal weight and smoking BN was associated with higher birth weight for gestational age (dose-response)	5
Romero-Martínez and Moya-Albiol 2014	Observational study	AN (*n* = 34), HCs (*n* = 40)	Maternal age, maternal BMI, right 2D:4D ratio, education, marital status of children	Levels of prenatal testosterone (indirectly studied by 2D:4D ratio)	–	–	Low 2D:4D ratio (higher prenatal testosterone) in AN. Salivary testosterone was negatively related to the 2D:4D ratio	3
Culbert et al. 2015	Michigan State University Twin Registry	Study 1 (2D:4D ratios): monozygotic (*n* = 229) and dyzigotic (*n* = 180) Study 2 (OS-F study): 1538 males and females and 131 non-twin females as an additional comparison group	–	Levels of prenatal testosterone (indirectly studied by 2D:4D ratio and by OS-F twin study)	–	–	In both studies, higher prenatal testosterone exposure (lower 2D:4D, females from opposite-sex twin pairs vs controls) predicted lower disordered eating symptoms in early adolescence and young adulthood	3
Lofrano-Prado et al. 2015	Cross-sectional observational study	College students (*n* = 408)	Mother’s age > 25 years		Number of obstetric complications, cesarean delivery	Low birth weight (< 2500 g), no breastfeeding, not first in the birth order	Mother’s age lower than 25 years old is associated with AN symptoms BN symptoms are associated with # of obstetric complications	4
Micali et al. 2015	Cohort study (very preterm cohort *n* = 476)	VPT, < 33 weeks (*n* = 143)	–	–	Cesarean section, vaginal delivery	Birthweight, gestational age, ventricular dilatation, neonatal complications	ED symptomatology was not associated with any of the factors. Cesarean delivery was associated with compensatory behaviors.	2
St-Hilaire et al. 2015	Cohort study (Project Ice Storm cohort, *n* = 54)	Teenagers born from cohort mothers (*n* = 54)	Maternal age, maternal education, social class, maternal stress exposure, trimester of stress exposure (Storm 24, IES-R scales)	–	–	Birth weight, length of gestation	Higher EAT-26 scores are associated with maternal exposure to stress in the third trimester	3
Tenconi et al. 2015	Birth cohort study (Padua new Birth Cohort 1969–1997 and previous 1971–1979)	New cohort: AN (*n* = 150), BN (*n* = 35), HCs (*n* = 73) Whole cohort: AN (*n* = 264), BN (*n* = 108), HCs (*n* = 624)	Maternal age, social class	Bleeding, preeclampsia, maternal diabetes, threatened miscarriages, and anemia	Inertia uteri, breech delivery, premature rupture of the membranes, breech delivery, placental infarction or abruption, placenta previa, meconium staining of the amniotic fluid, forceps or vacuum extraction, umbilical cord wrapped around the infant’s neck, and cephalopelvic disproportion, # of obstetric complications, need for resuscitation, O2 and intubation, cephalohematoma	Small for gestation age, birth weight, cyanosis, respiratory and cardiac problems, jaundice, neuromuscular disturbances such as hyporeactivity, hypotonia, and tremors, hypothermia	AN maintains its associations as shown in Favaro (2006), and higher maternal age and weight gain during pregnancy. Maternal diabetes and anemia lost their association BN is associated with neonatal hyporeactivity, early feeding difficulties, short and small for gestational age	5
Matinolli et al. 2016	Population cohort study (ESTER 1985–1989 e AYLS 1985–1986)	Early preterm (*n* = 185), late preterm (*n* = 348), term-born control (*n* = 637)	Maternal weight, maternal smoke habit, socioeconomic status, education	Gestational diabetes, hypertension, preeclampsia, eclampsia	–	Early preterm birth (< 32 weeks) and late preterm birth (32–37 weeks)	EDI-2 scores were significantly lower in early-born preterms than in HCs , in particular in “Body dissatisfaction” and “Drive for thinness” subscales	4
Sacks et al. 2016	Population cohort study (Soroka University Medical Center, Israel 1991–2014 (*n* = 231,271)	EDs (*n* = 486)	Maternal age, maternal obesity	Gestational diabetes mellitus (type A1 and type A2)	–	Gestational age at birth	ED and other psychiatric disorders in the offspring were associated with gestational diabetes mellitus	2
Su et al. 2016	Population-based cohort (Denmark 1973–2000 (*n* = 1,034,539) and Sweden 1970–1997 (*n* = 1,246,560)	AN (*n* = 5878), BN (*n* = 1722), mixed ED (*n* = 3159), HCs (*n* = 2110,755)	Maternal loss of a close relative 1 year prior to or during pregnancy	–	–	–	ED, BN, and mixed ED were associated with maternal exposure to prenatal loss	4
Razaz et al. 2018	Retrospective Swedish cohort study (1992–2002, *n* = 486,688)	AN (*n* = 2414)	Maternal BMI, years of education, maternal age at delivery, smoke habit	Multiparity	Vaginal delivery, vaginal instrumental delivery, elective cesarean section, emergency cesarean section	Birthweight for gestational age, gestational age at delivery	AN was associated with higher maternal age, higher maternal education, multiparity, and preterm birth The rate of AN decreased with maternal overweight and obesity in a dose-response manner	5

*AN* anorexia nervosa, *BN* bulimia nervosa, *HC* healthy control, *ED* eating disorder, *EDI-2* Eating Disorder Inventory-2, *EAT-26* Eating Attitude Test-26, *BITE* Bulimic Investigatory Test, *FSS* female same-sex twins, *FOS* female opposite-sex twins, *MOS* male opposite-sex twins, *MSS* male same-sex twins, *SS* same sex, *OS* opposite sex, *VPT* very preterm, *BMI* body mass index

As reported in Table 1, the methodological quality of each study was assessed by two investigators (F.C. and E.M.) using MMAT (Mixed Methods Appraisal Tool, 2018 version; Hong et al. 2018) that allows a quality assessment of qualitative, quantitative, and mixed methods studies (Pluye et al. 2009; Pace et al. 2012) with a double evaluation and a high intraclass correlation. The overall agreement was 90% on the MMAT items; when discrepancies emerged, they were resolved through discussion. The quality score ranged from 0 (no criteria met) to 5 (all criteria met).

According to earlier literature (Whittemore and Knafl 2005), due to the extreme heterogeneity of the study results, it has not been possible to conduct a meta-analysis so it was preferred to proceed with a thematic meta-synthetic approach to critically synthesize literature on the role of prenatal and perinatal factors on the onset of EDs. The findings of all the included studies were independently read and re-read, coded, and organized into categories, which were then compared across studies to identify relationships and themes (Whittemore and Knafl 2005).

## Results

Of the 815 studies screened, 96 full-text articles were assessed for eligibility, and 37 were finally included in the systematic review (please see Table 1 and Supplementary Materials for all details).

**Figure 2:**
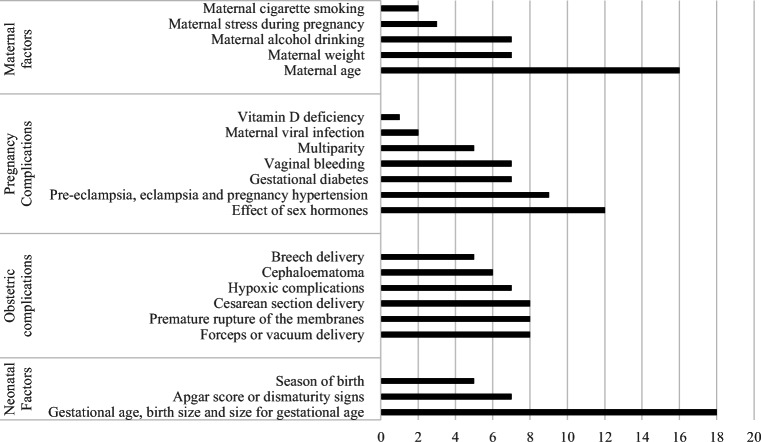
The numerosity of studies across categories of maternal factors, pregnancy complications, obstetric complications, and neonatal factors

The outcomes assessed in the studies were highly variable and were divided as follows: (1) maternal factors, (2) pregnancy complications, (3) obstetric complications, and (4) neonatal factors. Study distribution has been outlined in Fig. 2.

**Fig. 3:**
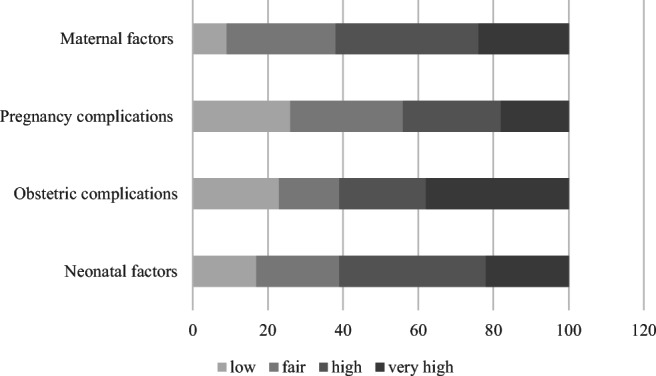
The methodological quality of the studies included in this review

As shown in Fig. 3, the methodological quality of studies ranged from low (2 points) to high (5 points). Overall, no studies were rated as showing very low methodological quality (1 point) and only the category of pregnancy complications showed the majority of studies (56%) as scoring low and fair. In fact, all other categories (pregnancy complications, obstetric complications, and neonatal factors) reported about 60% of studies with high (4 points) or very high (5 points) methodological quality scores (see Fig. 3).

The most frequent limitations were participants’ poor representativeness of the target population (Shoebridge and Gowers 2000; Foley et al. 2001; Feingold et al. 2002; Klump et al. 2006; Culbert et al. 2008, 2013; Smith et al. 2010; Coombs et al. 2011; Quinton et al. 2011; Nosarti et al. 2012; St-Hilaire et al. 2015), small sample size (Shoebridge and Gowers 2000; Feingold et al. 2002; Klump et al. 2006; Culbert et al. 2008, 2013; Smith et al. 2010; Quinton et al. 2011), and unappropriate measurements (e.g., self-report diagnosis, ED symptomatology investigated with questionnaires on healthy participants, lack of a clear DSM or ICD diagnosis; Feingold et al. 2002; Montgomery et al. 2005; Klump et al. 2006; Culbert et al. 2008, 2013, 2015; Baker et al. 2009; Nicholls and Viner 2009; Smith et al. 2010; Wehkalampi et al. 2010; Coombs et al. 2011; Quinton et al. 2011; Lydecker et al. 2012; Nosarti et al. 2012; Allen et al. 2013; Romero-Martínez and Moya-Albiol 2014; St-Hilaire et al. 2015; Lofrano-Prado et al. 2015; Matinolli et al. 2016; Sacks et al. 2016), and uncontrolled confounders (Shoebridge and Gowers 2000; Foley et al. 2001; Feingold et al. 2002; Culbert et al. 2008, 2013; Raevuori et al. 2008; Baker et al. 2009; Nicholls and Viner 2009; Smith et al. 2010; Wehkalampi et al. 2010; Coombs et al. 2011; Quinton et al. 2011; Vellisca et al. 2013; Micali et al. 2015; Sacks et al. 2016). MMAT scores were lower for cross-sectional and case-control (ranging from 2 to 4 points) than cohort studies (ranging from 3 to 5 points).

Notwithstanding the aforementioned weaknesses, some data gathered by the most robust studies should be acknowledged (see Table 2): in fact, mixed diagnoses of EDs were associated with maternal stress during pregnancy and preterm birth; still, BN was consistently associated with maternal psychosocial stress during pregnancy. Finally, multiple factors, according to the available data, resulted to be related to the onset of AN in the offspring: higher maternal age, preeclampsia and eclampsia, multiparity, hypoxic complications, prematurity or preterm birth (< 32 weeks), and being small for gestational birth size.

**Table 2:** Results summary of the studies included in this review grouped by diagnoses of eating disorders

	Eating disorders (EDs)	Anorexia nervosa (AN)	Bulimia nervosa (BN)
Factors supported by more robust evidence	•Maternal stress during pregnancy •Preterm birth	•Higher maternal age •Preeclampsia and eclampsia •Multiparity •Hypoxic complications •Prematurity or preterm birth (< 32 weeks) •Small for gestational age or lower birth size	•Maternal stress during pregnancy
Factors supported by less robust evidence	•Gestational diabetes •Vit. D deficiency	•Low maternal weight •Viral infection during pregnancy •Season of birth (spring)	•Maternal smoke habit •Vit. D deficiency •Dysmaturity signs

## Maternal factors

### Maternal age (16 studies)

A total of 16 studies assessed maternal age as a possible factor linked to the risk for AN or BN (Cnattingius et al. 1999; Feingold et al. 2002; Lindberg and Hjern 2003; Montgomery et al. 2005; Favaro et al. 2006, 2008; Wehkalampi et al. 2010; Nosarti et al. 2012; Allen et al. 2013; Goodman et al. 2014; Romero-Martínez and Moya-Albiol 2014; Tenconi et al. 2015; St-Hilaire et al. 2015; Lofrano-Prado et al. 2015; Sacks et al. 2016; Razaz and Cnattingius 2018). Five studies out of 16 found that higher maternal age was significantly associated with an increased risk of AN (Cnattingius et al. 1999; Lindberg and Hjern 2003; Goodman et al. 2014; Tenconi et al. 2015; Razaz and Cnattingius 2018). A register study found instead that adolescents with mothers who were young at the time of birth had a lower risk of developing AN compared with the adolescents of mothers who were 25–28 years old at birth (Lindberg and Hjern 2003).

### Maternal weight (7 studies)

Out of a total of 7 studies addressing maternal weight (Montgomery et al. 2005; Wehkalampi et al. 2010; Allen et al. 2013; Romero-Martínez and Moya-Albiol 2014; Matinolli et al. 2016; Sacks et al. 2016; Razaz and Cnattingius 2018), the majority did not show significant associations between maternal body mass index (BMI) and the onset of either AN or BN in the offspring. In contrast, a recent study found that the risks of AN in girls born at term decreased with maternal overweight and obesity in a dose-response manner (Razaz and Cnattingius 2018).

### Maternal cigarette smoking (2 studies) and alcohol drinking (7 studies)

Only 2 studies investigated maternal alcohol use and both failed to show any associations between maternal alcohol use and EDs onset in the offspring (Feingold et al. 2002; Allen et al. 2013). Seven studies investigated instead the role of maternal cigarette smoke (Montgomery et al. 2005; Nicholls and Viner 2009; Wehkalampi et al. 2010; Allen et al. 2013; Goodman et al. 2014; Matinolli et al. 2016; Razaz and Cnattingius 2018), reporting an association between maternal smoking and BN diagnosis in the offspring, even after controlling for confounding factors such as offspring BMI in adulthood or variation between childhood and adult BMI (Montgomery et al. 2005). In contrast, another study found a strong negative association between mother’s smoking and AN, but this was substantially attenuated upon adjustment for parental education (Goodman et al. 2014). Still, all the other studies did not identify an association (Nicholls and Viner 2009; Wehkalampi et al. 2010; Allen et al. 2013; Matinolli et al. 2016; Razaz and Cnattingius 2018).

### Maternal stress during pregnancy (3 studies)

Only 3 studies assessed maternal stress during pregnancy and EDs diagnosis in the offspring and all consistently found a significant association (Taborelli et al. 2013; St-Hilaire et al. 2015; Su et al. 2016). A large population-based cohort study found that girls who were born from mothers who lost a close relative from 1 year before the beginning of pregnancy to the whole duration of pregnancy had an increased risk of suffering from an EDs than healthy controls, with similar results for mixed EDs and BN, but not for AN (Su et al. 2016). Similarly, it has been observed that the daughters of mothers with chronic anxiety during pregnancy had an increased risk of AN (Taborelli et al. 2013). Additionally, maternal stress in the third trimester of pregnancy was associated with elevated scores on a screening tool for EDs (St-Hilaire et al. 2015).

### Pregnancy complications

#### Preeclampsia, eclampsia, pregnancy hypertension (9 studies), and maternal anemia (3 studies)

Nine studies investigated preeclampsia, eclampsia, pregnancy hypertension (Cnattingius et al. 1999; Foley et al. 2001; Feingold et al. 2002; Lindberg and Hjern 2003; Favaro et al. 2006, 2008; Tenconi et al. 2015; Matinolli et al. 2016), and three maternal anemia (Favaro et al. 2006; Nicholls and Viner 2009; Tenconi et al. 2015). Out of 9 studies, only 3 of them found that AN was significantly associated with preeclampsia (Lindberg and Hjern 2003; Favaro et al. 2006; Tenconi et al. 2015), while only in one work (Favaro et al. 2006) an association with maternal anemia during pregnancy was found. Nevertheless, the same group disconfirmed such an association in a more recent study (Tenconi et al. 2015).

#### Gestational diabetes (7 studies)

Seven studies assessed the role of gestational diabetes (Cnattingius et al. 1999; Favaro et al. 2006, 2008; Nicholls and Viner 2009; Tenconi et al. 2015; Matinolli et al. 2016; Sacks et al. 2016). Out of the available studies, only 2 found an association between gestational diabetes and EDs in the offspring, the first on a sample with mixed ED diagnoses (Sacks et al. 2016) and the latter on a sample with AN (Favaro et al. 2006). However, a more recent study did not confirm this datum (Tenconi et al. 2015).

#### Maternal viral infection (2 studies)

Two studies analyzed the association between in utero viral infection exposition, in particular rubella (Foley et al. 2001; Favaro et al. 2011) and chickenpox (Favaro et al. 2011). Only one study found that exposure to rubella or chickenpox during the sixth month of pregnancy was associated with an increased risk of developing AN in the offspring (Favaro et al. 2011).

#### Vitamin D deficiency (1 study)

One study assessed the role of vitamin D deficiency in pregnancy, finding that EDs were predicted by low maternal vitamin D level at 18-week pregnancy, even after controlling for family sociodemographic factors, BMI, and depressive symptoms (Allen et al. 2013). However, BN was the only disorder in which the risk remained significantly increased when EDs were assessed separately.

#### Effect of sex hormones (12 studies)

Six studies assessed prenatal androgen exposure using the 2D:4D ratio (Klump et al. 2006; Smith et al. 2010; Coombs et al. 2011; Quinton et al. 2011; Romero-Martínez and Moya-Albiol 2014; Culbert et al. 2015). In general, the 4th digit tends to be longer than the 2nd in males, whereas in females the 2nd and 4th digits tend to be of equal length (Berenbaum et al. 2009). Lower 2D:4D ratios (second finger shorter than the fourth finger) point to higher prenatal androgen exposure (Berenbaum et al. 2009). Two studies (Klump et al. 2006; Culbert et al. 2015) found that ED symptomatology [i.e., body dissatisfaction (Klump et al. 2006), weight preoccupation (Klump et al. 2006), binge eating (Klump et al. 2006), compensatory behaviors (Klump et al. 2006), and disordered eating (Culbert et al. 2015)] was associated with lower levels of prenatal testosterone. In males, greater prenatal testosterone exposure is associated with less disordered eating, less drive for leanness, but increased drive for muscularity (Smith et al. 2010). Two other studies found that AN was instead significantly associated with higher testosterone exposure during pregnancy (Quinton et al. 2011; Romero-Martínez and Moya-Albiol 2014), while BN was associated with lower levels of prenatal testosterone (Quinton et al. 2011).

Six studies indirectly investigated the role of prenatal sex hormone effects in opposite-sex twin cohorts (Culbert et al. 2008, 2013, 2015; Raevuori et al. 2008; Baker et al. 2009; Lydecker et al. 2012). The highest levels of disordered eating were both observed in same-sex and opposite-sex female twins (Culbert et al. 2008). In a subsequent study (Culbert et al. 2013), disordered eating was not associated with opposite-sex or same-sex twins in pre-early puberty. However, during later phases of puberty, females from opposite-sex twin pairs exhibited less restrictive eating behavioral patterns than females from same-sex twin pairs. Other three studies (Raevuori et al. 2008; Baker et al. 2009; Lydecker et al. 2012) did not support the hypothesis that having a female co-twin increases ED risk in either male or female twins, but another found that lifetime prevalence of AN was 1.6–3.3% in women from opposite-sex twin pairs, while it was 2.9–5.1% in women from same-sex pairs (Raevuori et al. 2008).

#### Multiparity (5 studies)

Out of 5 studies assessing the role of multiparity in the risk of EDs onset (Cnattingius et al. 1999; Feingold et al. 2002; Nosarti et al. 2012; Goodman et al. 2014; Razaz and Cnattingius 2018), only 2 of them found an association with later offspring diagnosis of AN (Goodman et al. 2014; Razaz and Cnattingius 2018).

#### Vaginal bleeding (7 studies)

Despite 7 studies assessing the role of vaginal bleeding during pregnancy, none of them found an association with offspring ED diagnosis/symptomatology (Cnattingius et al. 1999; Shoebridge and Gowers 2000; Foley et al. 2001; Feingold et al. 2002; Favaro et al. 2006, 2008; Tenconi et al. 2015).

### Obstetric complications

Three studies found that an increasing number of obstetric complications was correlated to a higher risk of EDs in the offspring, specifically AN (Foley et al. 2001; Favaro et al. 2006) and BN (Foley et al. 2001; Favaro et al. 2006; Lofrano-Prado et al. 2015), and a lower age of onset of the EDs (Favaro et al. 2006). Few obstetric complications have been associated with BN, but only one study found an association between bulimic symptoms in college students and the presence of any obstetric complications at birth (Lofrano-Prado et al. 2015).

#### Hypoxic complications (7 studies)

Seven studies assessed hypoxic complications, namely, umbilical cord wrapped around the neck, need for O2, and placental infarction (Foley et al. 2001; Feingold et al. 2002; Lindberg and Hjern 2003; Favaro et al. 2006, 2008; Nicholls and Viner 2009; Tenconi et al. 2015). As a result, only 3 studies found an association between hypoxic complications and AN (Lindberg and Hjern 2003; Favaro et al. 2006; Tenconi et al. 2015) or BN (Favaro et al. 2006).

#### Breech delivery (5 studies)

Out of 5 studies assessing breech delivery (Foley et al. 2001; Feingold et al. 2002; Lindberg and Hjern 2003; Favaro et al. 2006; Tenconi et al. 2015), only one study (Lindberg and Hjern 2003) found an association with later AN diagnosis.

#### Cephalohematoma (6 studies)

Cephalohematoma was investigated by 6 studies (Cnattingius et al. 1999; Lindberg and Hjern 2003; Favaro et al. 2006, 2008; Goodman et al. 2014; Tenconi et al. 2015) but only 2 of them found an association with AN diagnosis in the offspring (Cnattingius et al. 1999; Lindberg and Hjern 2003).

#### Premature rupture of the membranes (8 studies)

Eight studies assessed premature rupture of the membranes (Cnattingius et al. 1999; Foley et al. 2001; Feingold et al. 2002; Lindberg and Hjern 2003; Favaro et al. 2006, 2008; Goodman et al. 2014; Tenconi et al. 2015). Only one study found an association with AN (Lindberg and Hjern 2003) but was then disconfirmed by a later larger work (Goodman et al. 2014).

#### Cesarean section delivery (8 studies) and forceps or vacuum delivery (8 studies)

Eight studies investigated the role of cesarean section delivery (Cnattingius et al. 1999; Shoebridge and Gowers 2000; Foley et al. 2001; Feingold et al. 2002; Goodman et al. 2014; Micali et al. 2015; Lofrano-Prado et al. 2015; Razaz and Cnattingius 2018) and forceps or vacuum delivery (Cnattingius et al. 1999; Shoebridge and Gowers 2000; Foley et al. 2001; Favaro et al. 2006, 2008; Goodman et al. 2014; Tenconi et al. 2015; Razaz and Cnattingius 2018). Only one study found a weak evidence of an association with AN diagnosis (Goodman et al. 2014) while another (Micali et al. 2015) found that cesarean section was instead associated with compensatory behaviors.

### Neonatal factors

#### Gestational age, birth size, and size for gestational age (18 studies)

Eighteen studies assessed prematurity in terms of gestational age, birth size, or birth size for gestational age (Cnattingius et al. 1999; Shoebridge and Gowers 2000; Foley et al. 2001; Feingold et al. 2002; Lindberg and Hjern 2003; Favaro et al. 2006, 2008; Nicholls and Viner 2009; Wehkalampi et al. 2010; Nosarti et al. 2012; Allen et al. 2013; Goodman et al. 2014; Micali et al. 2015; Tenconi et al. 2015; St-Hilaire et al. 2015; Matinolli et al. 2016; Sacks et al. 2016; Razaz and Cnattingius 2018).

Gestational age (< 32 weeks) has been consistently found to be associated with EDs, even adjusting for confounders (Nosarti et al. 2012; Foley et al. 2001; Goodman et al. 2014; Cnattingius et al. 1999; Lindberg and Hjern 2003; Razaz and Cnattingius 2018). With more detail, low gestational age was associated with later AN diagnosis with an odds ratio ranging from 1.9 (95% CI 1.2–3.3; Lindberg and Hjern 2003) to 3.2 (95% CI 1.6–6.2; Cnattingius et al. 1999).

Three studies found that AN diagnosis was associated with being small for gestational age at birth (Cnattingius et al. 1999) or having a Ponderal Index < 25 (Favaro et al. 2006; Tenconi et al. 2015).

Concerning BN, two studies found that BN was associated with low birth weight for gestational age (Favaro et al. 2006; Tenconi et al. 2015); however, another study reported the opposite result (Goodman et al. 2014).

Two studies on unaffected individuals found that adolescents and young adults born preterm showed lower scores on eating psychopathology (i.e., drive for thinness, body dissatisfaction, and bulimia) than those who were not born preterm (Wehkalampi et al. 2010) even after controlling for confounders (Matinolli et al. 2016).

#### Apgar score or dysmaturity signs (7 studies)

Seven studies assessed Apgar score (Cnattingius et al. 1999; Shoebridge and Gowers 2000; Lindberg and Hjern 2003; Nosarti et al. 2012) or dysmaturity signs (Favaro et al. 2006, 2008; Tenconi et al. 2015) such as hypotonia, hyporeactivity, hypothermia, tremors, and feeding problems at birth. One study found that AN diagnosis was associated with low Apgar score at birth (Lindberg and Hjern 2003); in keeping with these findings, the other two studies found that hyporeactivity was a significant independent predictor of the development of AN even after adjusting for confounders (Favaro et al. 2006; Tenconi et al. 2015). Additionally, the same studies found neonatal hyporeactivity and early eating difficulties as adjusted risk factors for BN (Favaro et al. 2006; Tenconi et al. 2015). In another study, the presence of signs of neonatal dysmaturity influenced the development of high harm avoidance, a risk factor of EDs (Favaro et al. 2008).

#### Season of birth (5 studies)

Five studies assessed season or month of birth and the association with EDs providing consistent support to an association of AN with being born in spring (Morgan and Lacey 2000; Favaro et al. 2011; Vellisca et al. 2013; Winje et al. 2013; Allen et al. 2013).

## Discussion

The aim of this systematic review was to highlight the association between prenatal and perinatal factors and the subsequent development of EDs, investigating the hypothesis that these factors could impair neurodevelopment, similarly to the model proposed for schizophrenia (Geddes and Lawrie 1995; Verdoux et al. 1997; Geddes et al. 1999; Cannon et al. 2000; Clarke et al. 2011; Rapoport et al. 2012). When analyzing the main findings of this review, it should be also borne in mind that it has been brought to the surface the inconsistency of the available body of evidence on this topic and the lack of a robust framework able to explain the possible relationships between prenatal and perinatal factors and clinical variables in EDs. Nevertheless, some relevant main findings emerged as well: first, maternal stress during pregnancy and preterm birth emerged as the most supported factors impacting on a diagnosis of EDs in the offspring. Similarly, maternal stress during pregnancy was robustly associated with the onset of BN. Finally, the association between prenatal and perinatal factors and AN resulted to be complex and many-sided: in fact, higher maternal age, preeclampsia and eclampsia, multiparity, hypoxic complications, and prematurity or preterm birth (< 32 weeks) or being small for gestational age or with a low birth size were the most sound factors in the association with the onset of AN in the offspring.

That said, in keeping with the second aim of this work, future lines of research can be outlined in order to bridge these gaps in the literature: for example, no studies investigated binge eating disorder (BED) and further works may want to tackle the aforementioned methodological weaknesses (i.e., composite variable for prenatal and perinatal risk factors; lack of a shared definition of such risk factors) with ad hoc study designs. As a first step, as suggested by literature (Krug et al. 2013), all factors should be divided into four groups: pregnancy complications, labor and delivery complications, and fetal distress signs/neonatal complications. As a second step, other candidates proposed by this review could be added as well, including: mothers’ characteristics (i.e., age, smoke habit, weight, stressful events during pregnancy) and timing (seasonality). Doing so, it will be finally possible to conduct a meta-analysis of these data yielding quantitative results as well.

With more detail, the main findings for each category (i.e., maternal factors, pregnancy complications, obstetric complications, and neonatal factors) are described below.

### Maternal factors

With respect to maternal factors, maternal age has been deepened by a number of robust studies finding that a higher maternal age was significantly associated with an increased risk of AN in the offspring (Cnattingius et al. 1999; Feingold et al. 2002; Favaro et al. 2011; Goodman et al. 2014; Razaz and Cnattingius 2018). However, Lofrano-Prado and collaborators (Lofrano-Prado et al. 2015) found a contrasting result, observing that AN symptomatology in healthy students was 0.5 times lower for those students born from the oldest mothers (> 25 years old). This conflicting result may be due to the numerous possible confounding factors, such as the application of psychometric assessments to a non-clinical sample or a sociocultural selection bias. Physiopathogenesis underlying the association of the diagnosis of AN in the offspring and a higher maternal age is yet to be clarified and could be due both to a greater risk of pregnancy complications in older mothers or to later socio-educational and environmental factors linked to older mothers, so future studies are needed to clarify these matters.

Recent evidence indicates that another maternal factor, namely, obesity and metabolic diseases, may have a long-term impact on psychiatric conditions of the offspring, such as attention deficit hyperactive disorder, autism, and schizophrenia (Rivera et al. 2015). In contrast, in the field of EDs, only one study found that the risk of AN in girls born at term decreased with maternal overweight and obesity in a dose-response manner (Razaz and Cnattingius 2018). However, such a finding was not confirmed in the sibling control analyses, so other genetic or familiar environmental factors may be involved as well.

Out of seven studies available, maternal smoke was reported as significant only by two studies with contrasting results (Montgomery et al. 2005; Goodman et al. 2014). In fact, on one hand, a positive association with BN, even after controlling for confounding factors such as offspring BMI in adulthood or variation between childhood and adult BMI, was reported (Montgomery et al. 2005), but on the other hand, a negative association between mother’s smoking and AN was shown, even if attenuated upon adjustment for parental education (Goodman et al. 2014). Although a larger number of studies (i.e., 7) investigated the association between maternal alcohol use and EDs onset in the offspring, no significant data emerged; since this datum is not in line with other fields of psychiatry (Pagnin et al. 2019), future studies are needed to clarify this issue.

Interestingly, psychosocial stress was found to be strongly associated with both EDs and BN in the offspring, on the basis of all the available studies (i.e., 3) that supported this datum with robust evidence (Taborelli et al. 2013; St-Hilaire et al. 2015; Su et al. 2016). Also, maternal stress has been reported to entail a greater risk of impulsive and compensatory behaviors, as hypothesized also in other fields of psychiatry (Abbott et al. 2018). This line of research needs a deeper investigation because it could underlie an important link between endocrine functions, genetics, and temperamental traits. Stress activates the HPA axis, increasing the release of glucocorticoids (St-Hilaire et al. 2015) that could cross the maternal placenta and impact on the development of metabolism, fetal growth, and immune functions during pregnancy. Glucocorticoids could also affect the development of the fetal HPA axis, causing an alteration in stress-response mechanisms, emotional dysregulation, and increased risk for anxiety disorders and EDs in childhood and adulthood (Meyer and Hamel 2014; St-Hilaire et al. 2015).

#### Pregnancy complications

Concerning pregnancy complications, gestational diabetes could alter fetal neurodevelopment during critical periods exposing the fetus to elevated glucose levels (Georgieff 2006). Out of seven studies, two found an association between gestational diabetes and EDs in the offspring (Favaro et al. 2006; Sacks et al. 2016), although subsequent work partially disconfirmed such findings (Tenconi et al. 2015). Similarly, more research is needed also on the role of viral infection during pregnancy: only the study by Favaro et al. (2011) reported that exposure to rubella or chickenpox during the sixth month was associated with an increased risk of developing AN in the offspring.

Animal studies suggested that transient prenatal vitamin D deficiency is associated with altered brain development (Ali et al. 2018) and low maternal vitamin D during pregnancy was identified as a significant predictor of later schizophrenia (McGrath et al. 2010, 2011) and autism (Ali et al. 2018) in the offspring. Surprisingly, only one study was conducted in the ED field, reporting data in line with those of general psychiatry: in fact, EDs were predicted by low maternal vitamin D level at 18-week pregnancy, even after controlling for family sociodemographic factors, BMI, and depressive symptoms (Allen et al. 2013). However, BN was the only disorder in which the risk remained significantly increased when EDs were assessed separately, so no definitive conclusions can be drawn.

Exposure to testosterone during pregnancy has been associated with organizational permanent effects of eating behavior: in animal models, prenatal testosterone exposure increased food intake in male mammals, while in females low levels of testosterone were associated with later restrictive eating behavior (Donohoe and Stevens 1983; Madrid et al. 1993). Lower levels of prenatal testosterone exposure have been associated with body dissatisfaction, weight preoccupation, binge eating, and compensatory behaviors in females (Klump et al. 2006). It has been also suggested that the relatively low level of testosterone before birth in females permits their brains to respond to estrogens at puberty when the hormones activate the genes contributing to disordered eating in vulnerable girls (Klump et al. 2006). Prenatal exposure to male hormones could be indirectly investigated in adult females using the finger-length ratios (2D:4D), a sexually dimorphic trait that correlates with prenatal androgen exposure (Klump et al. 2006) or in opposite-sex twins cohorts, where the female fetus is exposed to higher levels of testosterone by sharing the womb with the male fetus (Resnick et al. 1993; Cohen-Bendahan et al. 2004, 2005). The hypothesis that higher prenatal testosterone exposure could increase food intake and protect against the development of disordered eating symptoms is intriguing although debated. Studies of 2D:4D ratios have yielded more positive evidence than those examining females from opposite-sex twin pairs, but the overall results are very controversial. Future studies should focus on direct assessment of sex hormone levels during pregnancy and perform a better confounder analysis on the other risk factors.

#### Obstetric complications

Obstetric complications that seem to have more robust evidence of association with later AN onset are hypoxic complications (Lindberg and Hjern 2003; Favaro et al. 2006; Tenconi et al. 2015), breech delivery (Lindberg and Hjern 2003), and cephalohematoma (Cnattingius et al. 1999; Lindberg and Hjern 2003). Preeclampsia and eclampsia, severe hypoxic and fetal hypoperfusion pregnancy complications, were associated with a later diagnosis of AN in three more robust- evidence cohort studies, two from the same expanded pool of patients (Lindberg and Hjern 2003; Favaro et al. 2006; Tenconi et al. 2015). Observation of neuropsychological deficits (Galderisi et al. 2003), subtle neurological abnormalities (Gillberg et al. 1994), and nonreversible morphological brain changes (Katzman et al. 1997; Chowdhury et al. 2003) might suggest that impairment in neurodevelopment could be one of the possible pathways for the development of an ED (Connan et al. 2003). Obstetric complications might have more than one role in their etiopathogenesis: they could cause hypoxia-induced damage to the brain thus impairing the neurodevelopment of the fetus (Cannon et al. 2000). Also, the adequacy of nutrition during pregnancy and the postnatal period could influence the adults’ nutritional status and their appetite programming throughout life (Jones et al. 2017). Perinatal hypoxia/ischemia could cause disturbances of the dopaminergic system that persists in adulthood and impairs the neurotrophic signaling critical for pre- and postnatal brain development (Giannopoulou et al. 2018).

Despite eight studies investigating the role of cesarean section delivery, only weak evidence is available on the association with AN diagnosis (Goodman et al. 2014) or ED symptomatology (Micali et al. 2015). Similarly, also data on the premature rupture of the membranes garnered an overall weak association with EDs.

#### Neonatal factors

Prematurity, in particular very preterm birth (≤ 32 weeks), or being small for gestational age and birth size have been associated with several cohort studies to AN (Cnattingius et al. 1999; Foley et al. 2001; Lindberg and Hjern 2003; Favaro et al. 2006; Goodman et al. 2014; Razaz and Cnattingius 2018) and EDs (Nosarti et al. 2012). Interestingly, a clear dose-response pattern has been found between lower gestational age and higher risk for AN, with a gradient observed even within the term (births at 37 weeks of gestation) (Goodman et al. 2014). Cnattingius et al. (1999) showed that among girls born very preterm, the risk of subsequent development of AN was higher among girls who were small for gestational age (OR 5.7, 95% CI 1.1–28.7) than among girls with higher birth weight for gestational age (OR 2.7, 95% CI 1.2–5.8). Less clear is the association between BN and birth weight: two cohort studies (Favaro et al. 2006; Tenconi et al. 2015) from the same population found that BN was associated with being born small for gestational age; however, another study found the opposite result (Goodman et al. 2014). Micali et al. (2015) despite not having found any associations between perinatal predictors and ED psychopathology, observed that those very preterm adults that at the age of 21 years presented with ED symptoms had a smaller gray matter volume in the posterior cerebellum and a smaller white matter volume in the fusiform gyrus bilaterally at the age of 14–15 years. Early alteration of the cerebellum sub-networks linked with somatosensory, interoceptive, and emotional processings was recently found (Gaudio et al. 2018). These findings, if confirmed by further studies, could help to explain the abnormal integration of somatosensory and homeostatic signals, which may lead to body image disturbances in AN.

Broadly speaking, premature newborns need to face a difficult environment that increases stress potentially influencing the psychic and brain development in turn generating epigenetic changes. Follow-up studies often report neurocognitive inabilities with multi-level minimal impairments (Fumagalli et al. 2018; Nist et al. 2019). In the same vein, predisposing factors for EDs could impact not only directly on the relationship with food but also indirectly (i.e., socio-emotional and/or neuropsychological difficulties) increasing individuals’ vulnerability to factors occurring later in the lifespan, for example, cultural factors and hormonal changes in adolescence.

The findings that healthy individuals born preterm scored lower on eating psychopathology than HCs born at term (Wehkalampi et al. 2010; Matinolli et al. 2016) are apparently in contrast with some aforementioned studies. Notwithstanding, these inconsistencies could be due to a sub-optimal diagnostic assessment (EDI-2 and not the ICD/DSM gold standard) or to a sample selection bias or to the lack of adjustment for confounders. Future studies on healthy individuals born preterm are warranted to better understand their eating style.

Interestingly, dysmaturity signs (e.g., hyporeactivity) have been linked to personality alterations in AN (Favaro et al. 2006; Tenconi et al. 2015) and BN (Favaro et al. 2006; Tenconi et al. 2015). As shown by Favaro et al. (2008), neonatal dysmaturity influences the development of particular temperamental dimensions (Cloninger et al. 1993), such as harm avoidance (HA), a temperament dimension that has been associated with a higher risk of AN onset (Atiye et al. 2015). HA reflects the tendency to respond intensely to aversive stimuli and involves anticipatory worry about possible problems (Favaro et al. 2008). It is considered a marker of emotional vulnerability to depression (Kampman and Poutanen 2011) not only because individuals with high HA are more anxiety-prone but also because of their limited ability to recover from depression.

Seasonality is still a controversial topic that could underlie many other factors, such as gestational vitamin D, exposure to infectious agents, temperature and weather, and/or pregnancy and birth complications, all of which have the potential to influence fetal or infant neurodevelopment. Results are mixed, with studies supporting the season of birth hypothesis for generic EDs (Eagles et al. 2001; Watkins et al. 2002; Waller et al. 2002), although this association is lost when both AN (Button and Aldridge 2007; Vellisca et al. 2013; Winje et al. 2013) and BN (Morgan and Lacey 2000; Button and Aldridge 2007) are studied separately. Given the several methodological issues affecting these data (e.g., different latitudes, the accuracy of diagnosis, sample sizes, and comparison groups), caution is required when reading these findings.

## Conclusions

The investigation of the association of prenatal and perinatal factors with later onset of psychiatric conditions is particularly hard to perform from a methodological standpoint. This is even more true when such an evaluation is applied to quite rare conditions like AN (Hoek 2006). Notwithstanding, the available body of evidence supports maternal stress during pregnancy and preterm birth as associated with the development of mixed diagnoses of EDs; still, BN was consistently associated with maternal psychosocial stress during pregnancy. Finally, multiple factors were reported to have an impact on the onset of AN in the offspring, namely, higher maternal age, preeclampsia and eclampsia, multiparity, hypoxic complications, prematurity, or preterm birth (< 32 weeks), and being small for gestational birth size. However, our review contributed also to shed light on the plethora of methodological inconsistencies and flaws that characterize these lines of research (e.g., inclusion biases, self-report assessments of the EDs, lack of a shared definition of prenatal/perinatal factors, to name a few), potentially generating new ideas on how to tackle these weaknesses and finally provide a meta-analysis on this clinically relevant topic. More studies on larger samples evaluating multiple factors with a longitudinal design—the most fruitful research methodology given the research questions—are necessary to draw more solid conclusions on this topic. Also, as reported earlier (Krug et al., 2013), a shared definition of risk factors is required; in this light, the classification system adopted in this review (i.e., maternal factors, pregnancy complications, obstetric complications, and neonatal factors) could be a starting point to promote the additional debate on prenatal and perinatal factors in the field of EDs in turn encouraging well-designed studies.

## Electronic supplementary material

ESM 1 (DOCX 441 kb)
