# Supplementary material for: The role of prenatal and perinatal factors in eating disorders: a systematic review
Source: Arch Womens Ment Health. 2020 Aug 7;24(2):185–204. doi: 10.1007/s00737-020-01057-5 (PMC7979621; doi:10.1007/s00737-020-01057-5)
Supplement: Supplementary file 1 — (DOCX 441 kb) [file 737_2020_1057_MOESM1_ESM.docx]

# The role of prenatal and perinatal factors in eating disorders: a systematic review

# **Abstract**

**Purpose:** Numerous studies showed that factors influencing fetal development and neonatal period could lead to lasting alterations in the brain of the offspring, in turn increasing the risk for eating disorders (EDs). This work aims to systematically and critically review the literature on the association of prenatal and perinatal factors with the onset of EDs in the offspring, updating previous findings and focusing on anorexia nervosa (AN) and bulimia nervosa (BN).

**Methods:** A systematic literature search was performed on Pubmed, PsycINFO, and Scopus. The drafting of this systematic review was conducted following the PRISMA statement criteria and the methodological quality of each study was assessed by MMAT 2018.

**Results:** A total of 37 studies were included in this review. Factors that showed a more robust association with AN were higher maternal age, pre-eclampsia and eclampsia, multiparity, hypoxic complications, prematurity, or being born preterm (<32 weeks) and small for gestational age or lower birth size. BN was only associated with maternal stress during pregnancy. Many methodological flaws emerged in the considered studies, so further research is needed to clarify these inconsistencies.

**Conclusion:** Altogether, data are suggestive of an association between prenatal and perinatal factors and the onset of EDs in the offspring. Nevertheless, given the methodological quality of the available literature, firm conclusions cannot be drawn and whether this vulnerability is specific to EDs or mental disorders remains to be defined. Also, a strong need for longitudinal and well-designed studies on this topic emerged.

**Funding:** No funding was associated with this study.

# **Keyword**

# Eating Disorders

# Anorexia nervosa

# Bulimia nervosa

# Pregnancy complications

# Obstetric complications

# **Introduction**

Eating disorders (EDs) are complex mental illnesses characterized by unknown etiology, with many putative risk factors (Fairburn and Harrison 2003; Dalle Grave 2011; Jacobi et al. 2011) increasing the risk of the ED onset. Genetic risk factors are of great importance, with genes impacting on the development of both anorexia nervosa (AN) and bulimia nervosa (BN) as well as on their predisposing traits (Trace et al. 2013; Baker et al. 2017). Altered neurodevelopment has been implicated in the pathogenesis of several mental disorders (Katzman et al. 1997; Chowdhury et al. 2003; King et al. 2018) such as schizophrenia (Geddes and Lawrie 1995; Verdoux et al. 1997; Geddes et al. 1999; Cannon et al. 2000), attention deficit hyperactivity disorder (Lindström et al. 2006), and autism (Gardener et al. 2009). With more detail, hypoxic complications and prematurity have been associated with schizophrenia risk (Geddes and Lawrie 1995; Verdoux et al. 1997; Geddes et al. 1999; Cannon et al. 2000), leading to the formulation of a ‘neurodevelopmental hypothesis for schizophrenia’ (Rapoport et al. 2012).

Several studies (Gillberg et al. 1994; Connan et al. 2003) linked prenatal and perinatal complications (Raevuori et al. 2014) to EDs, with the hypothesis that subtle neurological damages (Gillberg et al. 1994), neuropsychological disabilities (Galderisi et al. 2003), and nonreversible morphological brain changes could ease the disorder onset. Krug and coworkers (Krug et al. 2013) systematically reviewed the literature on obstetric complications (OCs) and EDs, selecting 14 articles, and performed a meta-analysis where possible. Conflicting results emerged, so their meta-analysis found a non-significant association between instrumental delivery and prematurity and EDs risk. More recently, a descriptive review of 22 articles focusing on additional risk factors (e.g., the role of sex hormones, maternal status, and maternal EDs) found once more mixed results (Raevuori et al. 2014). Another review of 13 studies (Jones et al. 2017) focused instead on “fetal programming” as a model on how stimuli/insults occurring during critical or sensitive periods of fetal development could have physiological effects that unfold across the lifespan. The latter review introduced new risk factors, such as maternal stress during pregnancy, but finding again controversial results. Therefore, the aim of this systematic review is two-fold: a) to critically review updated literature on the association of prenatal and perinatal factors with the onset of EDs in the offspring, and b) to expand knowledge on the critical points that need to be addressed by future lines of research.

#

# **Methods**

# **Search strategy and selection criteria**

The drafting of this systematic review was conducted following the PRISMA statement criteria (Moher et al. 2009), and the studies included in this systematic review have been evaluated by MMAT (Mixed Methods Appraisal Tool, 2018 version; Hong et al. 2018).

# A systematic literature search was done between March 1, and May 1, 2019, including online database searches, namely Pubmed, PsycINFO and Scopus, and journal hand searching to ensure a wide inclusion of eligible studies.

# The search was designed to include those studies published since 1998; this is a reasoned choice as the purpose of this systematic review was to analyze the most recent studies on the topic. All full-text studies published were included if they met the following inclusion criteria:

- Study type criteria: cohort study and case-control study
- Eating Disorder assessment criteria: EDs: DSM (III, III-R, IV, IV-TR, 5) diagnosis, ICD (8,9,10) diagnosis, clinical diagnosis or self-report of diagnosis or EDs symptomatology obtained with questionnaires scores;
- Risk assessment criteria: obstetric birth records, parents recall and/or a combination of both;

The following list of terms were included and combined together in different search lines: “eating disorders”, “anorexia nervosa”, “bulimia nervosa”, “pregnancy complications”, “obstetric complications”, “prenatal risk factors”, “perinatal risk factors”, “gestational diabetes”, “hypertensive disease in pregnancy”, “maternal anaemia”, “pre-eclampsia”, “eclampsia”, “maternal viral infection”, “season of birth”, “vit. D levels during pregnancy”, “prenatal sex hormones”, “dismaturity”, “preterm birth”, “mother weight”, “mother smoke habit”, “maternal stress”, “maternal anxiety”, “placenta previa”, “pregnancy bleeding”, “breech delivery”, “induced labour”, “inertia uteri”, “premature rupture of the membrane”, “forceps”, “caesarean section”, “vaginal instrumental delivery”, “vacuum extraction”, “cephalhaematoma”, “umbilical cord wrapped around the neck”, “placental infarction”, “cyanosis”, “jaundice”, “need for resuscitation”, “need for oxygen”, “need for intubation”, “birth weight”, “prematurity”, “tremors”, “hypothermia”, “hypotonia” and “neuromuscular disturbances”*.*

# Two investigators (EM, FC) screened the title and abstracts of 851 studies identified through the search using the inclusion criteria. Full-text articles were retrieved for all studies that met the inclusion criteria or required more information than was provided in the abstract. The reasons for excluding the studies were documented and shown in Figure 1.


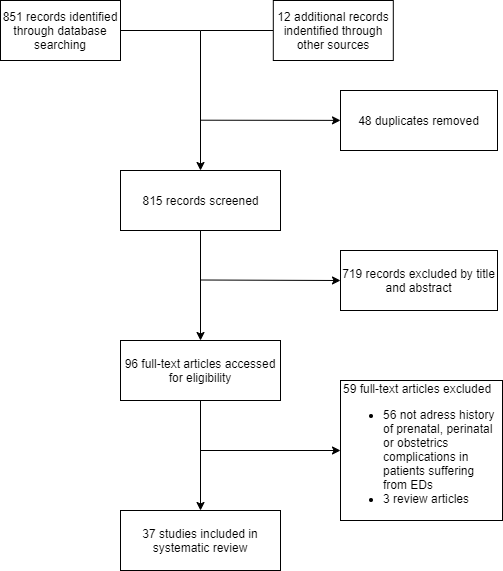


**Figure 1 –** Study selection process

# **Data analysis**

All database search results were imported into Zotero. Duplicate entries were removed before the screening. Data from the studies were extracted and summarized (see Table 1 and Supplementary Materials). Data extracted included authors, year, study type, sample size, psychiatric assessment, maternal factors, pregnancy complications, obstetric complications, neonatal factors, and main findings.

As reported in Table 1, the methodological quality of each study was assessed by two investigators (FC and EM) using MMAT (Mixed Methods Appraisal Tool, 2018 version; Hong et al. 2018) that allows a quality assessment of qualitative, quantitative and mixed methods studies (Pluye et al. 2009; Pace et al. 2012) with a double evaluation and a high intraclass correlation. The overall agreement was 90% on the MMAT items; when discrepancies emerged, they were resolved through discussion. The quality score ranged from zero (no criteria met) to five (all criteria met).

According to earlier literature (Whittemore and Knafl 2005), due to the extreme heterogeneity of studies results, it has not been possible to conduct a meta-analysis so it was preferred to proceed with a thematic metasynthetic approach to critically synthesize literature on the role of prenatal and perinatal factors on the onset of EDs. The findings of all the studies were independently read and re-read, coded, and organized into categories, which were then compared across studies to identify relationships and themes (Whittemore and Knafl 2005).

**Results**

Of the 815 studies screened, 96 full-text articles were assessed for eligibility, and 37 were finally included in the systematic review (please see Table 1 and Supplementary Materials for all details).

**Table 1 – Articles summary of study results included in this review.**

|  |  |  | **Assessed conditions** | | | |  |  |
| --- | --- | --- | --- | --- | --- | --- | --- | --- |
| **Authors** | **Study Type** | **Sample** | **Maternal Factors** | **Pregnancy Complications** | **Obstetric Complications** | **Neonatal Factors** | **Main findings** | **Quality Rating** |
|  |  |  |  |  |  |  |  |  |
| Cnattingius et al. 1999 | Case-Control Study with randomly selected control (Swedish Inpatient Register – 1973-1984) | AN (n=781), HC (n=3905) | Maternal age, | Hypertensive disease, diabetes, bleeding during pregnancy, multiparity | Inertia uteri, forceps, or vacuum delivery, preterm rupture of the membranes, cesarean section, cephalhematoma, other trauma | Gestational age, birth weight and birth weight for gestational age, Apgar Score, jaundice, | AN diagnosis was associated with maternal age, very preterm birth ( < or = 32 weeks), small for gestational age in very preterm birth, cephalhaematoma | 5 |
| Morgan et al. 2000 | Case-Control Study | BN (n=935), patients with AN history and BN diagnosis (n=227) general population data | - | - | - | Month of birth | BN diagnosis is not associated with a specific month of birth  AN history and BN diagnosis is associated with a peak season of birth in March | 4 |
| Shoebridge & Gowers et al. 2000 | Case-control study | AN (n=40) and HC (n=40) | - | Antepartum hemorrhage, | Toxaemia, previous obstetric complications, forceps delivery, cesarean section | Mean birth weight, gestation length (full-term, 36 weeks, 32 weeks) APGAR score, the baby looked after in a special care baby unit | No association was found | 2 |
| Foley et al. 2001 | Twin Cohort Study (Virginia Twin Registry, n=2352) | AN (n=7), broadly defined AN (bdAN) (n=71), BN (n=42), broadly defined BN (bdBN) (n=100), twin control (n=1,586) | - | High blood pressure, vaginal bleeding, seizures or toxemia, german measles, any other complication, any prenatal -complication | Premature contractions, labor lasting more than 24hr, breech delivery, cesarean delivery, forceps delivery, cord wrapped around the neck, blue at birth, required an incubator, other perinatal complications, any perinatal complication | Birth weight, gestational age, jaundice, failure to breath at first, convulsions, blood transfusion, | AN: low gestational age, # prenatal maternal complications  bdAN: prenatal maternal complications  BN: # prenatal maternal complications  bdBN: # prenatal maternal complications | 3 |
| Feingold et al. 2002 | HIstorical Prospective Cohort (Thomas Jefferson University Hospital 1979-1981, n=86) | Infants born preterm (N=84) | Mother older than 40 years, alcohol abuse | Pre-eclampsia, eclampsia, chorioamnionitis, urinary tract infection, cervicitis, asthma, hypertension, hyperemesis gravidarum, cervical cancer, vaginal bleeding, multiparity | Premature rupture of membranes, cesarean delivery, breech or transverse delivery, abruptio placentae, placenta previa, incompetent cervix, the total number of complications | Small for gestational age, | No association with EDs symptomatology for any of the variables studied | 2 |
|  |  |  |  |  |  |  |  |  |
| Lindberg et al. 2003 | Population Cohort Study (Swedish Register Data 1973-1982 n=989,871) | AN (n=1122), HC (n=988,749) | Maternal age | Preeclampsia | Premature rupture of the membranes placental abruption, breech delivery, cephalhaematoma, neonatal O2 necessity | Gestational age, size for gestational age, prematurity, Apgar score, distress | AN diagnosis was associated with maternal age (>25 years), gestational age at birth (23-32 weeks), preeclampsia, premature rupture of membranes, low Apgar, O2 necessity, and distress, cephaloematoma, breech delivery | 4 |
| Montgomery et al. 2005 | Cohort Study (British Cohort Study 1970 n=4046) | BN (n=100), HC (3946) | Maternal age at delivery, smoke habit, occupation, social class, psychiatric morbidity, BMI | - | - | - | Self-reported diagnosis of BN is associated with maternal smoke during pregnancy | 3 |
| Favaro et al. 2006 | Birth Cohort Study, (Padua Birth Cohort 1971-1979) | AN (n=114), BN (n=73),  HC (n=554) | Maternal age, social class, | Bleeding, preeclampsia, maternal  diabetes, threatened miscarriages and anemia | Inertia uteri, breech delivery, premature rupture of the membranes, breech delivery, placental infarction or abruption, placenta previa, meconium staining of the amniotic fluid, forceps or vacuum extraction, the umbilical cord wrapped around the infant’s neck, and cephalopelvic need for resuscitation O2, and intubation, disproportion, # of obstetric complications, cephalhaematoma | Small for gestation age, birth weight, cyanosis, respiratory and cardiac problems, jaundice, neuromuscular disturbances such as hypo-reactivity, hypotonia, and tremors, hypothermia, | AN diagnosis was associated with diabetes, anemia, preeclampsia, placental infarction, ponderal index <25, neonatal cardiac problems, neonatal hyporeactivity, # of complications, the umbilical cord wrapped around neck  BN was associated with neonatal hypo-reactivity, early feeding difficulties, placental infarction, low birth weight for gestational age, # of complications (p<0.01) | 5 |
| Klump et al. 2006 | Twin Cohort Study (Michigan State Twin Study) | FSS (n=113) | - | Levels of prenatal testosterone (indirectly studied by 2D:4D ratio) | - | - | ED symptomatology was associated with lower levels of prenatal testosterone (higher 2D:4D ratio) | 2 |
| Culbert et al. 2008 | Twin Cohort Study (Michigan State Twin Study) | FSS (n=304), FOS (n=59), MOS (n=54), MSS (n=165), HC (n=69) | - | Levels of prenatal testosterone (indirectly studied by OS and SS twin study) | - | - | Highest levels of disordered eating were observed for fSS twins, followed by fOS twins, mOS twins, and mSS twins | 3 |
| Favaro et al. 2008 | Birth Cohort Study, (Padua Birth Cohort 1971-1979) | AN (n=66), BN (n=44), HC (n=257) | Maternal age | Vaginal bleeding, preeclampsia, maternal  diabetes, threatened miscarriages and anemia | Inertia uteri, breech delivery, premature rupture of the membranes, placental infarction or abruption, placenta previa, meconium staining of the amniotic fluid, forceps or vacuum extraction, the umbilical cord wrapped around the infant’s neck, cephalhaematoma, need for resuscitation O2 and intubation. | Small for gestational age, birth weight, cyanosis, respiratory and cardiac problems, jaundice, neuromuscular disturbances such as hypo-reactivity, hypotonia, and tremors, hypothermia | Preterm birth and neonatal dysmaturity were associated with high harm avoidance in the offspring affected by EDs | 4 |
| Raevuori et al. 2008 | Twin Cohort Study (FinnTwin 16, 1975-1979 sample n=2426) | 2426 female twins with known zigosity (OS dizygotic n=793, SS dizygotic n=765, monozygotic n=868), 1962 male twins (OS dizygotic n=717, SS dizygotic n=705, monozygotic n=540) | - | Levels of prenatal testosterone (indirectly studied by OS and SS twin study) | - | - | Opposite-sex twin pairs were not significantly different from monozygotic or same-sex dizygotic twins (female) in the association with AN or BN. | 4 |
| Baker et al. 2009 | Swedish Twin Study of Child and Adolescent Development (TCHAD) 1985-1986 | 439 identical females, 213 fraternal females, 461 identical males, 344 fraternal males, 371 opposite-gender twin pairs | - | Levels of prenatal testosterone (indirectly studied by twin study) | - | - | ED symptomatology was not associated with twin types | 3 |
| Nicholls et al. 2009 | Prospective Birth Cohort (British Cohort Study, n=16,567) | AN (n=101), HC (n=11,261) | Smoke habit, ethnicity, | Gestational diabetes, maternal anemia | Perinatal hypoxia | Birth weight, gestational age, prematurity (<37 weeks), | No significant differences between AN and HC | 3 |
| Smith et al. 2010 | Cross-sectional observational Study | Male college students (n=204) | - | Levels of prenatal testosterone (indirectly studied by 2D:4D ratio) | - | - | Higher testosterone exposure (lower 2D:4D ratio) was associated with less EDs symptoms, greater drive for muscularity, diminished drive for leanness | 2 |
| Wehkalampi et al. 2010 | Case-Control Study (Helsinki Study of Very Low Birth Weight Adults 1978-1985 (n=255) | Very Low Birth Weight Adults (n=255), Term (n=189) | Maternal age, smoke habit, BMI, education | - | - | Gestational age, very preterm birth (<32 weeks), very low birth weight – VLBW (<1500 g) | In both sexes, EDI-2 scores were lower in VLBW individuals than in controls | 3 |
| Coombs et al. 2011 | Observational study on a Midlands High School (UK) students | 132 pupils (age 11-14) | - | Levels of prenatal testosterone (indirectly studied by 2D:4D ratio) | - | - | No strong association was found | 2 |
| Favaro et al. 2011 | Birth Cohort Study, (Padua Birth Cohort 1970-1984, n=27,682) | AN (n=402), HC (n=26,950) | Maternal education, maternal, and social class | Maternal exposure during pregnancy to chickenpox, measles, rubella or influenza | - | Month of birth | AN diagnosis is associated with the exposure to rubella and chickenpox at the 6th of pregnancy.  Being born in June is associated with AN diagnosis. | 4 |
| Quinton et al. 2011 | Case-Control Study | AN (n=25), BN (n=26), HC (n=99) | - | Levels of prenatal testosterone (indirectly studied by 2D:4D ratio) | - | - | AN is associated with low 2D:4D ratio (higher prenatal testosterone)  BN: high 2D:4D ratio is associated with lower prenatal testosterone | 2 |
| Nosarti et al. 2012 | Historical population-based cohort study (Swedish Birth Register 1973-1985 and Hospital Discharge Register n= 1,301,522) | ED (n=997), other psychiatric disorder and HCs | Maternal age, maternal education maternal psychiatric family history | Parity | - | Gestational age, birth weight for gestational age, newborn sex, Apgar score at 5 min | ED: gestational week (<32 weeks) | 4 |
| Lydecker et al. 2013 | Three twin Cohort Study (MATR (US), NIPHTP (Norway), STAGE (Sweden)) | US: OS (n=481), SS (n=1,022)  Norway: OS (n=345), SS (n=1,430)  Sweden: OS (n=2,433), SS (n=7,000) | - | Levels of prenatal testosterone (indirectly studied by OS and SS twin study) | - | - | No association between co-twin sex and EDs. | 4 |
|  |  |  |  |  |  |  |  |  |
| Allen et al. 2013 | Population Cohort (Western Australian Pregnancy Cohort (Raine) n=2900) | ED (n=98), HC (n=428) | Maternal age, BMI, drinking alcohol, smoking cigarettes, education, family income | Serum 25(OH)D level at 18 weeks, kidney disease or dysfunction, urinary tract infection, thyroid dysfunction | - | Gestational age at birth, season of birth, weight, preterm birth (<37 weeks), newborn sex | EDs were associated with low quartile serum 25(OH)D level and season of birth (spring), kidney disease or dysfunction, sex (female)  BN was associated with lowquartile serum 25(OH)D level and season of birth (spring) | 3 |
| Culbert et al. 2013 | Twin Cohort Study (Michigan State Twin Study) | FSS, fOS, mOS, mSS (n=394), HC (n=63) | - | Levels of prenatal testosterone (indirectly studied by OS and SS twin study) | - | - | No differences were observed in levels of disordered eating attitudes in opposite-sex and same-sex twins in pre-early puberty.  During later phases of puberty, females from opposite-sex twin pairs exhibited lower disordered eating attitudes than females from same-sex twin pairs | 3 |
|  |  |  |  |  |  |  |  |  |
| Taborelli et al. 2013 | Case-Control Study (Sister Pair Study) | AN (n=94), BN (n=63), HC (n=157) | Anxiety during pregnancy (questionnaire) |  |  | - | Anxiety during pregnancy was associated with AN, but not BN | 4 |
| Vellisca et al. 2013 | Case-Control Study | AN (n=210), general population data | - | - | - | Month of birth | AN diagnosis is not associated with the month of birth | 4 |
| Winje et al. 2013 | Case-Control Study | AN (n=4045), HC with the same year of birth, sex, and region of birth | - | - | - | Month of birth | No significant differences between AN and HCs | 4 |
| Goodman et al. 2014 | Population Cohort Study (Swedish Register Data, 1975-1998, n=2,135,279) | AN (n=7,351), BN (n=2,804), other eating disorders (n=10,408), HC (n=2,015,862) | Maternal age, maternal education, number of full siblings, number of half-siblings, eating disorder in mother, multiple, smoke habit | Multiparity, | Premature rupture of membranes, cesarean section, instrumental delivery, cephalohematoma, other birth trauma | Gestational age, birth weight for gestational age birth length for gestational age, APGAR score | AN is associated with maternal age, multi-parity, lower gestational age (dose response). Weak evidence of cesarean and instrumental delivery and birth trauma other than cephalohematoma.  AN is negatively associated with higher maternal weight and smoking  BN is associated with higher birth weight for gestational age (dose-response) | 5 |
| Romero-Martínez et al. 2014 | Observational Study | AN (n=34), HC (n=40) | Maternal age, maternal BMI, Right 2D:4D ratio. Education, marital status, ° of children | Levels of prenatal testosterone (indirectly studied by 2D:4D ratio) | - | - | Low 2D:4D ratio (higher prenatal testosterone) in AN. Salivary testosterone is negatively related to the 2D:4D ratio. | 3 |
| Culbert et al. 2015 | Michigan State University Twin Registry | Study 1 (2D:4D ratios): Monozygotic (n=229) and dyzigotic (n=180)    Study 2 (OS-F study): 1538 males and females and 131 non-twin females as an additional comparison group | - | Levels of prenatal testosterone (indirectly studied by 2D:4D ratio and by OS-F twin study) | - | - | In both studies, higher prenatal testosterone exposure (lower 2D:4D, females from opposite-sex twin pairs vs controls) predicted lower disordered eating symptoms in early adolescence and young adulthood | 3 |
| Lofrano-Prado et al. 2015 | Cross-sectional observational study | College students (n=408) | Mother’s age >25 years |  | Number of obstetric complications, cesarean delivery | Low birth weight (<2500g), no breastfeeding, not first in the birth order | Mother’s age lower than 25 years old is associated with AN symptoms  BN symptoms are associated with # of obstetric complications | 4 |
| Micali et al. 2015 | Cohort Study (Very Preterm Cohort n=476) | VPT, <33 weeks, (n=143) | - | - | Cesarean section, vaginal delivery, | Birthweight, gestational age, ventricular dilatation, neonatal complications | ED symptomatology was not associated with any of the factors. Cesarean delivery was associated with compensatory behaviours. | 2 |
| St-Hilaire et al. 2015 | Cohort Study (Project Ice Storm Cohort, n=54) | Teenagers born from cohort mothers (n=54) | Maternal age, maternal education, social class, maternal stress exposure, trimester of stress exposure (Storm 24, IES-R scales), | - | - | Birth weight, length of gestation | Higher EAT-26 scores are associated with maternal exposure to stress in the third trimester | 3 |
| Tenconi et al. 2015 | Birth Cohort Study, (Padua new Birth Cohort 1969-1997 and previous 1971-1979) | New cohort: AN (n=150), BN (n=35), HC (n=73)  Whole cohort: AN (n=264), BN (n=108), HC (n=624) | Maternal age, social class, | Bleeding, preeclampsia, maternal  diabetes, threatened miscarriages and anemia | Inertia uteri, breech delivery, premature rupture of the membranes, breech delivery, placental infarction or abruption, placenta previa, meconium staining of the amniotic fluid, forceps or vacuum extraction, umbilical cord wrapped around the infant’s neck, and cephalopelvic disproportion, # of obstetric complications, need for resuscitation O2, and intubation, cephalhaematoma | Small for gestation age, birth weight, cyanosis, respiratory and cardiac problems, jaundice, neuromuscular disturbances such as hypo-reactivity, hypotonia, and tremors, hypothermia | AN maintains its associations as shown as Favaro, 2006, and higher maternal age and weight gain during pregnancy. Maternal diabetes and anemia lost their association.  BN is associated with neonatal hypo-reactivity, early feeding difficulties, short and small for gestational age | 5 |
| Mattinolli et al. 2016 | Population Cohort Study (ESTER 1985-1989 e AYLS 1985-1986) | Early preterm (n=185), late preterm (n=348), term-born control (n=637) | Maternal weight, maternal smoke habit, socioeconomic status, education | Gestational diabetes, hypertension, pre-eclampsia, eclampsia, | - | Early preterm birth (<32 weeks) and late preterm birth (32-37 weeks). | EDI-2 scores were significantly lower in early-born preterms than in controls, in particular in “Body dissatisfaction” and “Drive for thinness” subscales | 4 |
| Sacks et al. 2016 | Population Cohort Study (Soroka University Medical Center, Israel 1991-2014 (n=231,271) | EDs (n=486) | Maternal age, maternal obesity | Gestational diabetes mellitus (type A1 and type A2) | - | Gestational age at birth | ED and other psychiatric disorders in the offspring were associated with gestational diabetes mellitus | 2 |
| Su et al. 2016 | Population based cohort (Denmark 1973-2000 (n=1,034,539) and Sweden 1970-1997 (n=1,246,560)) | AN (n=5,878), BN (n=1,722), mixed ED (n= 3,159), HC (n=2,110,755) | Maternal loss or that of a close relative 1 year prior to or during pregnancy | - | - | - | ED, BN, mixed ED were associated with maternal exposure to prenatal loss | 4 |
| Razaz et al. 2018 | Retrospective Swedish cohort study (1992-2002, n= 486,688) | AN (n=2,414) | Maternal BMI, years of education, maternal age at delivery, smoke habit | Multiparity | Vaginal delivery, vaginal instrumental delivery, elective cesarean section, emergency cesarean section | Birth-weight for gestational age, gestational age at delivery | AN is associated with higher maternal age, higher maternal education, multiparity, and preterm birth.  The rate of AN decreased with maternal overweight and obesity in a dose-response manner | 5 |

*AN* anorexia nervosa, *BN* bulimia nervosa, *HC* healthy controls, *ED* eating disorder, *EDI-2* Eating Disorder Inventory, *EAT-26* Eating Attitude Test, *BITE* Bulimic Investigatory Test, *MBAS* Male Body Attitude Test, *EDEQ-4* Eating Disorder Examination Questionnaire, *MEBS* Minnesota Eating Behaviors Survey, *FSS* female same sax twins, *FOS* female opposite-sex twins, *MOS* male opposite-sex twins, *MSS* male same sec twins, *SS* same sex, *OS* opposite sex, *CNS* central nervous system, *VPT* very preterm, *BMI* body mass index.

## The outcomes assessed in the studies were highly variable and were divided as follows: 1) maternal factors, 2) pregnancy complications, 3) obstetric complications, and 4) neonatal factors. Study distribution has been outlined in Figure 2.

## **Figure 2. The numerosity of studies across categories of maternal factors, pregnancy complications, obstetric complications, and neonatal factors.**

###

## As shown in Figure 3, the methodological quality of studies ranged from low (2 points) to high (5 points). Overall, no studies were rated as showing very low methodological quality (1 point) and only the category of pregnancy complications showed the majority of studies (56%) as scoring low and fair. In fact, all other categories (pregnancy complications, obstetric complications, and neonatal factors) reported about 60% of studies with high (4 points) or very high (5 points) methodological quality scores (see Figure 3).

## **Figure 3. The methodological quality of the studies included in this review.**

The most frequent limitations were: participants’ poor representativeness of the target population (Shoebridge and Gowers 2000; Foley et al. 2001; Feingold et al. 2002; Klump et al. 2006; Culbert et al. 2008, 2013; Smith et al. 2010; Coombs et al. 2011; Quinton et al. 2011; Nosarti et al. 2012; St-Hilaire et al. 2015), small sample size (Shoebridge and Gowers 2000; Feingold et al. 2002; Klump et al. 2006; Culbert et al. 2008, 2013; Smith et al. 2010; Quinton et al. 2011), unappropriate measurements (e.g., self-report diagnosis, ED symptomatology investigated with questionnaires on healthy participants, lack of a clear DSM or ICD diagnosis; Feingold et al. 2002; Montgomery et al. 2005; Klump et al. 2006; Culbert et al. 2008, 2013, 2015; Baker et al. 2009; Nicholls and Viner 2009; Smith et al. 2010; Wehkalampi et al. 2010; Coombs et al. 2011; Quinton et al. 2011; Lydecker et al. 2012; Nosarti et al. 2012; Allen et al. 2013; Romero-Martínez and Moya-Albiol 2014; St-Hilaire et al. 2015; Lofrano-Prado et al. 2015; Matinolli et al. 2016; Nahum Sacks et al. 2016), and uncontrolled confounders (Shoebridge and Gowers 2000; Foley et al. 2001; Feingold et al. 2002; Culbert et al. 2008, 2013; Raevuori et al. 2008; Baker et al. 2009; Nicholls and Viner 2009; Smith et al. 2010; Wehkalampi et al. 2010; Coombs et al. 2011; Quinton et al. 2011; Vellisca et al. 2013; Micali et al. 2015; Sacks et al. 2016). MMAT scores were lower for cross-sectional and case-control (ranging from 2 to 4 points) than cohort studies (ranging from 3 to 5 points).

Notwithstanding the aforementioned weaknesses, some data gathered by the most robust studies should be acknowledged (see Table 2): in fact, mixed diagnoses of EDs were associated with maternal stress during pregnancy and preterm birth; still, BN was consistently associated with maternal psychosocial stress during pregnancy. Finally, multiple factors, according to the available data, resulted to be related to the onset of AN in the offspring: higher maternal age, pre-eclampsia and eclampsia, multiparity, hypoxic complications, prematurity or preterm birth (<32 weeks), and being small for gestational birth size.

**Table 2 - Results summary of the studies included in this review grouped by diagnoses of Eating Disorders.**

|  | **Eating Disorders (EDs)** | **Anorexia Nervosa (AN)** | **Bulimia Nervosa (BN)** |
| --- | --- | --- | --- |
| **Factors supported by more robust evidence** | - Maternal stress during pregnancy - Preterm birth | - Higher maternal age - Pre-eclampsia and eclampsia - Multiparity - Hypoxic Complications - Prematurity or Preterm Birth (<32 weeks) - Small for gestational age or lower birth size | - Maternal stress during pregnancy |
| **Factors supported by less robust evidence** | - Gestational Diabetes - Vit. D deficiency | - Low maternal weight - Viral infection during pregnancy - Season of birth (spring) | - Maternal smoke habit - Vit. D deficiency - Dismaturity signs |

### **Maternal factors**

### ***Maternal age (16 studies)***

### A total of 16 studies assessed maternal age as a possible factor linked to the risk for AN or BN (Cnattingius et al. 1999; Feingold et al. 2002; Lindberg and Hjern 2003; Montgomery et al. 2005; Favaro et al. 2006, 2008; Wehkalampi et al. 2010; Nosarti et al. 2012; Allen et al. 2013; Goodman et al. 2014; Romero-Martínez and Moya-Albiol 2014; Tenconi et al. 2015; St-Hilaire et al. 2015; Lofrano-Prado et al. 2015; Sacks et al. 2016; Razaz and Cnattingius 2018). Five studies out of 16 found that higher maternal age was significantly associated with an increased risk of AN (Cnattingius et al. 1999; Lindberg and Hjern 2003; Goodman et al. 2014; Tenconi et al. 2015; Razaz and Cnattingius 2018). A register study found instead that adolescents with mothers who were young at the time of birth had a lower risk of developing AN compared with the adolescents of mothers who were 25–28 years old at birth. (Lindberg and Hjern 2003)

###

### ***Maternal weight (7 studies)***

Out of a total of 7 studies addressing maternal weight (Montgomery et al. 2005; Wehkalampi et al. 2010; Allen et al. 2013; Romero-Martínez and Moya-Albiol 2014; Matinolli et al. 2016; Sacks et al. 2016; Razaz and Cnattingius 2018), the majority did not show significant associations between maternal body mass index (BMI) and the onset of either AN or BN in the offspring. In contrast, a recent study found that the risks of AN in girls born at term decreased with maternal overweight and obesity in a dose–response manner (Razaz and Cnattingius 2018).

***Maternal cigarette smoking (2 studies) and alcohol drinking (7 studies)***

Only 2 studies investigated maternal alcohol use and both failed to show any associations between maternal alcohol use and EDs onset in the offspring (Feingold et al. 2002; Allen et al. 2013). Seven studies investigated instead the role of maternal cigarette smoke (Montgomery et al. 2005; Nicholls and Viner 2009; Wehkalampi et al. 2010; Allen et al. 2013; Goodman et al. 2014; Matinolli et al. 2016; Razaz and Cnattingius 2018), reporting an association between maternal smoking and BN diagnosis in the offspring, even after controlling for confounding factors such as offspring BMI in adulthood or variation between childhood and adult BMI (Montgomery et al. 2005). In contrast, another study found a strong negative association between mother’s smoking and AN, but this was substantially attenuated upon adjustment for parental education (Goodman et al. 2014). Still, all the other studies did not identify an association (Nicholls and Viner 2009; Wehkalampi et al. 2010; Allen et al. 2013; Matinolli et al. 2016; Razaz and Cnattingius 2018).

***Maternal stress during pregnancy (3 studies)***

Only 3 studies assessed maternal stress during pregnancy and EDs diagnosis in the offspring and all consistently found a significative association (Taborelli et al. 2013; St-Hilaire et al. 2015; Su et al. 2016). A large population-based cohort study found that girls that were born from mothers who lost a close relative from one year before the beginning of pregnancy to the whole duration of pregnancy had an increased risk of suffering from an EDs than healthy controls, with similar results for mixed EDs and BN, but not for AN (Su et al. 2016). Similarly, it has been observed that the daughters of mothers with chronic anxiety during pregnancy had an increased risk of AN (Taborelli et al. 2013). Additionally, maternal stress in the third trimester of pregnancy was associated with elevated scores on a screening tool for EDs (St-Hilaire et al. 2015).

### **Pregnancy complications**

***Pre-eclampsia, eclampsia, pregnancy hypertension (9 studies,) and maternal anemia (3 studies)***

Nine studies investigated pre-eclampsia, eclampsia, pregnancy hypertension (Cnattingius et al. 1999; Foley et al. 2001; Feingold et al. 2002; Lindberg and Hjern 2003; Favaro et al. 2006, 2008; Tenconi et al. 2015; Matinolli et al. 2016) and three maternal anemia. (Favaro et al. 2006; Nicholls and Viner 2009; Tenconi et al. 2015). Out of 9 studies, only 3 of them found that AN was significantly associated with pre-eclampsia (Lindberg and Hjern 2003; Favaro et al. 2006; Tenconi et al. 2015), while only in one work (Favaro et al. 2006) an association with maternal anemia during pregnancy was found. Nevertheless, the same group disconfirmed such an association in a more recent study (Tenconi et al. 2015).

***Gestational diabetes (7 studies)***

Seven studies assessed the role of gestational diabetes (Cnattingius et al. 1999; Favaro et al. 2006, 2008; Nicholls and Viner 2009; Tenconi et al. 2015; Matinolli et al. 2016; Sacks et al. 2016). Out of the available studies, only 2 found an association between gestational diabetes and EDs in the offspring, the first on a sample with mixed ED diagnoses (Sacks et al. 2016) the latter on a sample with AN (Favaro et al. 2006). However, a more recent study did not confirm this datum (Tenconi et al. 2015).

***Maternal viral infection (2 studies)***

Two studies analyzed the association between in utero viral infection exposition, in particular rubella (Foley et al. 2001; Favaro et al. 2011) and chickenpox (Favaro et al. 2011). Only one study found that exposure to rubella or chickenpox during the sixth month of pregnancy was associated with an increased risk of developing AN in the offspring (Favaro et al. 2011).

***Vitamin D deficiency (1 study)***

One study assessed the role of vitamin D deficiency in pregnancy, finding that EDs were predicted by low maternal vitamin D level at 18-week pregnancy, even after controlling for family sociodemographic factors, BMI and depressive symptoms (Allen et al. 2013). However, BN was the only disorder in which the risk remained significantly increased when EDs were assessed separately.

***Effect of sex hormones (12 studies)***

Six studies assessed prenatal androgen exposure using the 2D:4D ratio (Klump et al. 2006; Smith et al. 2010; Coombs et al. 2011; Quinton et al. 2011; Romero-Martínez and Moya-Albiol 2014; Culbert et al. 2015). In general, the 4th digit tends to be longer than the 2nd in males, whereas in females the 2nd and 4th digits tend to be of equal length (Berenbaum et al. 2009). Lower 2D:4D ratios (second finger shorter than the fourth finger) points to higher prenatal androgen exposure (Berenbaum et al. 2009). Two studies (Klump et al. 2006; Culbert et al. 2015) found that ED symptomatology [i.e., body dissatisfaction (Klump et al. 2006), weight preoccupation (Klump et al. 2006), binge-eating (Klump et al. 2006), compensatory behaviors (Klump et al. 2006), and disordered eating (Culbert et al. 2015)] was associated with lower levels of prenatal testosterone. In males, greater prenatal testosterone exposure is associated with less disordered eating, less drive for leanness, but increased drive for muscularity (Smith et al. 2010). Two other studies found that AN was instead significantly associated with higher testosterone exposure during pregnancy (Quinton et al. 2011; Romero-Martínez and Moya-Albiol 2014), while BN was associated with lower levels of prenatal testosterone (Quinton et al. 2011).

Six studies indirectly investigated the role of prenatal sex hormones effects in opposite-sex twins cohorts (Culbert et al. 2008, 2013, 2015; Raevuori et al. 2008; Baker et al. 2009; Lydecker et al. 2012). The highest levels of disordered eating were both observed in same-sex and opposite-sex female twins (Culbert et al. 2008). In a subsequent study (Culbert et al. 2013), disordered eating was not associated with opposite-sex or same-sex twins in pre-early puberty. However, during later phases of puberty, females from opposite-sex twin pairs exhibited less restrictive eating behavioral patterns than females from same-sex twin pairs. Other three studies (Raevuori et al. 2008; Baker et al. 2009; Lydecker et al. 2012) did not support the hypothesis that having a female co-twin increases EDs risk in either male or female twins, but another found that lifetime prevalence of AN was 1.6–3.3% in women from opposite-sex twin pairs, while it was 2.9–5.1% in women from same-sex pairs (Raevuori et al. 2008).

### ***Multiparity (5 studies)***

### Out of 5 studies assessing the role of multiparity in the risk of EDs onset (Cnattingius et al. 1999; Feingold et al. 2002; Nosarti et al. 2012; Goodman et al. 2014; Razaz and Cnattingius 2018), only 2 of them found an association with later offspring diagnosis of AN (Goodman et al. 2014; Razaz and Cnattingius 2018).

### ***Vaginal bleeding (7 studies)***

### Despite 7 studies assessed the role of vaginal bleeding during pregnancy, none of them found an association with offspring EDs diagnosis/symptomatology (Cnattingius et al. 1999; Shoebridge and Gowers 2000; Foley et al. 2001; Feingold et al. 2002; Favaro et al. 2006, 2008; Tenconi et al. 2015).

### **Obstetric complications**

### Three studies found that an increasing number of obstetric complications correlates to a higher risk of EDs in the offspring, specifically AN (Foley et al. 2001; Favaro et al. 2006) and BN (Foley et al. 2001; Favaro et al. 2006; Lofrano-Prado et al. 2015), and a lower age of onset of the EDs (Favaro et al. 2006). Few obstetric complications have been associated with BN, but only one study found an association between bulimic symptoms in college students and the presence of any obstetric complications at birth (Lofrano-Prado et al. 2015).

***Hypoxic complications (7 studies)***

Seven studies assessed hypoxic complications, namely umbilical cord wrapped around the neck, need for O2, and placental infarction (Foley et al. 2001; Feingold et al. 2002; Lindberg and Hjern 2003; Favaro et al. 2006, 2008; Nicholls and Viner 2009; Tenconi et al. 2015). As a result, only 3 studies found an association between hypoxic complications and AN (Lindberg and Hjern 2003; Favaro et al. 2006; Tenconi et al. 2015) or BN (Favaro et al. 2006).

***Breech delivery (5 studies)***

Out of 5 studies assessing breech delivery (Foley et al. 2001; Feingold et al. 2002; Lindberg and Hjern 2003; Favaro et al. 2006; Tenconi et al. 2015) only one study (Lindberg and Hjern 2003) found an association with later AN diagnosis.

***Cephalohematoma (6 studies)***

Cephalohematoma was investigated by 6 studies (Cnattingius et al. 1999; Lindberg and Hjern 2003; Favaro et al. 2006, 2008; Goodman et al. 2014; Tenconi et al. 2015) but only 2 of them found an association with AN diagnosis in the offspring (Cnattingius et al. 1999; Lindberg and Hjern 2003).

***Premature rupture of the membranes (8 studies)***

Eight studies assessed premature rupture of the membranes (Cnattingius et al. 1999; Foley et al. 2001; Feingold et al. 2002; Lindberg and Hjern 2003; Favaro et al. 2006, 2008; Goodman et al. 2014; Tenconi et al. 2015). Only one study found an association with AN (Lindberg and Hjern 2003) but was then disconfirmed by a later larger work (Goodman et al. 2014).

***Cesarean section delivery (8 studies) and forceps or vacuum delivery (8 studies)***

Eight studies investigated the role of cesarean section delivery (Cnattingius et al. 1999; Shoebridge and Gowers 2000; Foley et al. 2001; Feingold et al. 2002; Goodman et al. 2014; Micali et al. 2015; Lofrano-Prado et al. 2015; Razaz and Cnattingius 2018) and forceps or vacuum delivery (Cnattingius et al. 1999; Shoebridge and Gowers 2000; Foley et al. 2001; Favaro et al. 2006, 2008; Goodman et al. 2014; Tenconi et al. 2015; Razaz and Cnattingius 2018). Only one study found weak evidence of an association with AN diagnosis (Goodman et al. 2014) while another (Micali et al. 2015) found that cesarean section was instead associated with compensatory behaviours.

## **Neonatal Factors**

***Gestational age, birth size and size for gestational age (18 studies)***

Eighteen studies assessed prematurity in terms of gestational age, birth size or birth size for gestational age (Cnattingius et al. 1999; Shoebridge and Gowers 2000; Foley et al. 2001; Feingold et al. 2002; Lindberg and Hjern 2003; Favaro et al. 2006, 2008; Nicholls and Viner 2009; Wehkalampi et al. 2010; Nosarti et al. 2012; Allen et al. 2013; Goodman et al. 2014; Micali et al. 2015; Tenconi et al. 2015; St-Hilaire et al. 2015; Matinolli et al. 2016; Sacks et al. 2016; Razaz and Cnattingius 2018).

Gestational age (< 32 weeks) has been consistently found as associated with EDs, even adjusting for confounders (Nosarti et al. 2012; Foley et al. 2001; Goodman et al. 2014; Cnattingius et al. 1999; Lindberg and Hjern 2003; Razaz and Cnattingius 2018). With more detail, low gestational age was associated with later AN diagnosis with an odds ratio ranging from 1.9 (95% CI 1.2-3.3;(Lindberg and Hjern 2003) to 3.2 (95% CI 1.6-6.2; (Cnattingius et al. 1999).

Three studies found that AN diagnosis was associated with being small for gestational age at birth (Cnattingius et al. 1999) or having a ponderal index <25 (Favaro et al. 2006; Tenconi et al. 2015).

Concerning BN, two studies found that BN was associated with low birth weight for gestational age (Favaro et al. 2006; Tenconi et al. 2015); however, another study reported the opposite result (Goodman et al. 2014).

Two studies on unaffected individuals found that adolescents and young adults born preterm showed lower scores on eating psychopathology (i.e., drive for thinness, body dissatisfaction, and bulimia) than those who were not born preterm (Wehkalampi et al. 2010) even after controlling for confounders (Matinolli et al. 2016).

***Apgar score or dismaturity signs (7 studies)***

Seven studies assessed Apgar score (Cnattingius et al. 1999; Shoebridge and Gowers 2000; Lindberg and Hjern 2003; Nosarti et al. 2012) or dismatury signs (Favaro et al. 2006, 2008; Tenconi et al. 2015) such as hypotonia, hyporeactivity, hypothermia, tremors and feeding problems at birth. One study found that AN diagnosis was associated with low Apgar score at birth (Lindberg and Hjern 2003); in keeping with these findings, other two studies found that hyporeactivity was a significant independent predictor of the development of AN even after adjusting for confounders (Favaro et al. 2006; Tenconi et al. 2015). Additionally, the same studies found neonatal hyporeactivity and early eating difficulties as adjusted risk factors for BN (Favaro et al. 2006; Tenconi et al. 2015). In another study, the presence of signs of neonatal dysmaturity influenced the development of high harm avoidance, a risk factor of EDs (Favaro et al. 2008).

***Season of birth (5 studies)***

Five studies assessed season or month of birth and the association with EDs providing consistent support to an association of AN with being born in spring (Morgan and Lacey 2000; Favaro et al. 2011; Vellisca et al. 2013; Winje et al. 2013; Allen et al. 2013).

#

# **Discussion**

The aim of this systematic review was to highlight the association of prenatal and perinatal factors on the subsequent development of EDs, investigating the hypothesis that these factors could impair neurodevelopment, similarly to the model proposed for schizophrenia (Geddes and Lawrie 1995; Verdoux et al. 1997; Geddes et al. 1999; Cannon et al. 2000; Clarke et al. 2011; Rapoport et al. 2012). When analyzing the main findings of this review, it should be also borne in mind that it has been brought to the surface the inconsistency of the available body of evidence on this topic and the lack of a robust framework able to explain the possible relationships between prenatal and perinatal factors and clinical variables in EDs. Nevertheless, some relevant main findings emerged as well: first, maternal stress during pregnancy and preterm birth emerged as the most supported factors impacting on a diagnosis of EDs in the offspring. Similarly, maternal stress during pregnancy was robustly associated with the onset of BN. Finally, the association between prenatal and perinatal factors and AN resulted to be complex and many-sided: in fact, higher maternal age, pre-eclampsia and eclampsia, multiparity, hypoxic complications, and prematurity or preterm birth (<32 weeks) or being small for gestational age or with a low birth size were the most sound factors in the association with the onset of AN in the offspring.

That said, in keeping with the second aim of this work, future lines of research can be outlined in order to bridge these gaps in the literature: for example, no studies investigated binge eating disorder (BED; APA-5) and further works may want to tackle the aforementioned methodological weaknesses (i.e., composite variable for prenatal and perinatal risk factors; lack of a shared definition of such risk factors) with ad hoc study designs. As a first step, as suggested in the literature (Krug et al. 2013), all factors should be divided into four groups: pregnancy complications, labor and delivery complications, and fetal distress signs/neonatal complications. As a second step, other candidates proposed by this review could be added as well, including: mothers’ characteristics (i.e., age, smoke habit, weight, stressful events during pregnancy) and timing (seasonality). Doing so, it will be finally possible to conduct a meta-analysis of these data yielding quantitative results as well.

With more detail, the main findings for each category (i.e., maternal factors, pregnancy complications, obstetric complications, and neonatal factors) are described below.

***Maternal factors***

With respect to maternal factors, maternal age has been deepened by a number of robust-evidence studies finding that higher maternal age was significantly associated with an increased risk of AN in the offspring (Cnattingius et al. 1999; Feingold et al. 2002; Favaro et al. 2011; Goodman et al. 2014; Razaz and Cnattingius 2018). However, Lofrano-Prado and collaborators (2015) found a contrasting result, observing that AN symptomatology in healthy students was 0.5 times lower for those students born from the oldest mothers (>25 years old). This conflicting result may be due to numerous possible confounding factors, such as the application of psychometric assessments to a non-clinical sample or a socio-cultural selection bias. Physiopathogenesis underlying the association of the diagnosis of AN in the offspring and a higher maternal age is yet to be clarified and could be due both to a greater risk of pregnancy complications in older mothers or to later socio-educational and environmental factors linked to older mothers so future studies are needed to clarify these matters.

Recent evidence indicates that another maternal factor, namely obesity and metabolic diseases may have a long-term impact on psychiatric conditions of the offspring, such as attention deficit hyperactive disorder, autism, and schizophrenia (Rivera et al. 2015). In contrast, in the field of EDs, only one study found that the risk of AN in girls born at-term decreased with maternal overweight and obesity in a dose–response manner (Razaz and Cnattingius 2018). However, such a finding was not confirmed in the sibling control analyses, so other genetic or familiar environmental factors may be involved as well.

Out of seven studies available, maternal smoke was reported as significant only by two studies with contrasting results (Montgomery et al. 2005; Goodman et al. 2014). In fact, on one hand, a positive association with BN, even after controlling for confounding factors such as offspring BMI in adulthood or variation between childhood and adult BMI, was reported (Montgomery et al. 2005) but on the other hand, a negative association between mother’s smoking and AN was shown, even if attenuated upon adjustment for parental education (Goodman et al. 2014). Although a larger number of studies (i.e., 7) investigated the association between maternal alcohol use and EDs onset in the offspring, no significant data emerged; since this datum is not in line with other fields of psychiatry, (Pagnin et al. 2019) future studies are needed to clarify this issue.

Interestingly, psychosocial stress was found to be strongly associated to both EDs and BN in the offspring, on the basis of all the available studies (i.e., 3) that supported this datum with robust evidence (Taborelli et al. 2013; St-Hilaire et al. 2015; Su et al. 2016). Also, maternal stress has been reported to entail a greater risk of impulsive and compensatory behaviors, as hypothesized also in other fields of psychiatry (Abbott et al. 2018). This line of research needs a deeper investigation because it could underlie an important link between endocrine functions, personality genetics, and temperamental traits. Stress activates the HPA axis, increasing the release of glucocorticoids (St-Hilaire et al. 2015) that could cross the maternal placenta and impact on the development of metabolism, fetal growth, and immune functions during pregnancy. Glucocorticoids could also affect the development of the fetal HPA axis, causing an alteration in stress-response mechanisms, emotional dysregulation, and increased risk for anxiety disorders and EDs in childhood and adulthood (Meyer and Hamel 2014; St-Hilaire et al. 2015).

***Pregnancy complications***

Concerning pregnancy complications, gestational diabetes could alter fetal neurodevelopment during critical periods exposing the fetus to elevated glucose levels (Georgieff 2006). Out of seven studies, two found an association between gestational diabetes and EDs in the offspring (Favaro et al. 2006; Sacks et al. 2016), although subsequent work partially disconfirmed such findings (Tenconi et al. 2015). Similarly, more research is needed also on the role of viral infection during pregnancy: only the study by Favaro and coworkers (2011) reported that exposure to rubella or chickenpox during the sixth month was associated with an increased risk of developing AN in the offspring.

Animal studies suggested that transient prenatal vitamin D deficiency is associated with altered brain development (Ali et al. 2018) and low maternal vitamin D during pregnancy was identified as a significant predictor of later schizophrenia (McGrath et al. 2010, 2011) and autism (Ali et al. 2018) in the offspring. Surprisingly, only one study was conducted in the ED field, reporting data in line with those of general psychiatry: in fact, EDs were predicted by low maternal vitamin D level at 18-week pregnancy, even after controlling for family sociodemographic factors, BMI and depressive symptoms (Allen et al. 2013). However, BN was the only disorder in which the risk remained significantly increased when EDs were assessed separately so no definitive conclusions can be drawn.

Exposure to testosterone during pregnancy has been associated with organizational permanent effects of eating behaviour: in animal models, prenatal testosterone exposure increased food intake in male mammals, while in females, low levels of testosterone were associated with later restrictive eating behaviour (Donohoe and Stevens 1983; Madrid et al. 1993). Lower levels of prenatal testosterone exposure have been associated with body dissatisfaction, weight preoccupation, binge eating, and compensatory behaviors in females (Klump et al. 2006). It has been also suggested that the relatively low level of testosterone before birth in females permits their brains to respond to estrogens at puberty when the hormones activate the genes contributing to disordered eating in vulnerable girls (Klump et al. 2006). Prenatal exposure to male hormones could be indirectly investigated in adult females using the finger-length ratios (2D:4D), a sexually dimorphic trait that correlates with prenatal androgen exposure (Klump et al. 2006) or in opposite-sex twins cohorts, where the female fetus is exposed to higher levels of testosterone by sharing the womb with the male fetus (Resnick et al. 1993; Cohen-Bendahan et al. 2004, 2005). The hypothesis that higher prenatal testosterone exposure could increase food intake and protect against the development of disordered eating symptoms is very suggestive, although studies are not fully consistent. Studies of 2D:4D ratios have yielded more positive evidence than those examining females from opposite-sex twin pairs, but overall results are very controversial. Future studies should focus on direct assessment of sex-hormones levels during pregnancy and perform a better confounders analysis on other risk factors.

***Obstetric complications***

Obstetric complications that seem to have more robust evidence of association with later AN onset are hypoxic complications (Lindberg and Hjern 2003; Favaro et al. 2006; Tenconi et al. 2015) breech delivery (Lindberg and Hjern 2003) and cephalohematoma (Cnattingius et al. 1999; Lindberg and Hjern 2003). Pre-eclampsia and eclampsia, severe hypoxic and fetal hypoperfusion pregnancy complications, were associated with a later diagnosis of AN in three more robust-evidence cohort studies, two from the same expanded pool of patients (Lindberg and Hjern 2003; Favaro et al. 2006; Tenconi et al. 2015). Observation of neuropsychological deficits (Galderisi et al. 2003), subtle neurological abnormalities (Gillberg et al. 1994), and nonreversible morphological brain changes (Katzman et al. 1997; Chowdhury et al. 2003) might suggest that impairment in neurodevelopment could be one of the possible pathways for the development of an ED (Connan et al. 2003). Obstetric complications might have more than one role in their etiopathogenesis: they could cause hypoxia-induced damage to the brain that impairs the neurodevelopment of the fetus (Cannon et al. 2000). Also, the adequacy of nutrition during pregnancy and the postnatal period could influence the adults’ nutritional status and their appetite programming throughout life (Jones et al. 2017). Perinatal hypoxia/ischemia could cause disturbances of the dopaminergic system that persists in adulthood and impairs the neurotrophic signaling critical for pre- and postnatal brain development (Giannopoulou et al. 2018).

Despite eight studies investigated the role of cesarean section delivery, only weak evidence is available on the association with AN diagnosis (Goodman et al. 2014) or EDs symptomatology (Micali et al. 2015). Similarly, also data on the premature rupture of the membranes garnered an overall weak association with EDs.

***Neonatal factors***

Prematurity, in particular very preterm birth (≤32 weeks), or being small for gestational age and birth size have been associated by several cohort studies to AN (Cnattingius et al. 1999; Foley et al. 2001; Lindberg and Hjern 2003; Favaro et al. 2006; Goodman et al. 2014; Razaz and Cnattingius 2018) and EDs (Nosarti et al. 2012). Interestingly, a clear dose-response pattern has been found between lower gestational age and higher risk for AN, with a gradient observed even within the term (births at 37 weeks of gestation) (Goodman et al. 2014). Cnattingius and coworkers (1999) showed that among girls born very preterm, the risk of subsequent development of AN was higher among girls who were small for gestational age (OR 5.7, 95% CI 1.1-28.7) than among girls with higher birth weight for gestational age (OR 2.7, 95% CI 1.2-5.8). Less clear is the association between BN and birth weight: two cohort studies (Favaro et al. 2006; Tenconi et al. 2015) from the same population found that BN was associated with being born small for gestational age; however, another study found the opposite result (Goodman et al. 2014). Micali and coworkers (2015) despite not having found any associations between perinatal predictors and ED psychopathology, observed that those very preterm adults that at age of 21 years presented with ED symptoms had a smaller grey matter volume in the posterior cerebellum and a smaller white matter volume in the fusiform gyrus bilaterally at the age of 14-15 years. Early alteration of cerebellum sub-networks linked with somatosensory, interoceptive, and emotional processings was recently found (Gaudio et al. 2018). These findings, if confirmed by further studies, could help to explain the abnormal integration of somatosensory and homeostatic signals, which may lead to body image disturbances in AN.

Broadly speaking, premature newborns need to face a difficult environment able to increase stress and to influence the psychic and brain development in turn generating epigenetic changes. Follow-up studies often report neurocognitive inabilities with multi-level minimal impairments (Fumagalli et al. 2018; Nist et al. 2019). In the same vein, predisposing factors for EDs could impact not only directly on the relationship with food but also indirectly (i.e., socio-emotional and/or neuropsychological difficulties) increasing individuals’ vulnerability to factors occurring later in the lifespan, for example, cultural factors and hormonal changes in adolescence.

The findings that healthy individuals born preterm scored lower on eating psychopathology than born at-term healthy individuals (Wehkalampi et al. 2010; Matinolli et al. 2016) is apparently in contrast with some aforementioned studies. Notwithstanding, these inconsistencies could be due to a sub-optimal diagnostic assessment (EDI-2 and not the ICD/DSM gold standard) or to a sample selection bias or to the lack of adjustment for confounders. Future studies on healthy individuals born preterm are warranted to better understand their eating style.

Interestingly, dismatury signs (e.g., hyporeactivity) have been linked to personality alterations in AN (Favaro et al. 2006; Tenconi et al. 2015) and BN (Favaro et al. 2006; Tenconi et al. 2015). As shown by Favaro and coworkers (2008) neonatal dysmaturity influence the development of particular temperamental dimensions (Cloninger et al. 1993), such as harm avoidance (HA), a temperament dimension that has been associated with a higher risk of AN onset (Atiye et al. 2015). HA reflects a tendency to respond intensely to aversive stimuli and involves anticipatory worry about possible problems (Favaro et al. 2008). It is considered a marker of emotional vulnerability to depression (Kampman and Poutanen 2011) not only because individuals with high HA are more anxiety-prone, but also because of their limited ability to recover from depression.

Seasonality is still a controversial topic that could underlie many other factors, such as gestational vitamin D, exposure to infectious agents, temperature and weather, and/or pregnancy and birth complications, all of which have the potential to influence fetal or infant neurodevelopment. Results are mixed, with studies supporting the season of birth hypothesis for generic EDs (Eagles et al. 2001; Watkins et al. 2002; Waller et al. 2002), although this association is lost when both AN (Button and Aldridge 2007; Vellisca et al. 2013; Winje et al. 2013) and BN (Morgan and Lacey 2000; Button and Aldridge 2007) are studied separately. Given the several methodological issues affecting these data (e.g., different latitudes, the accuracy of diagnosis, sample sizes, and comparison groups) caution is required when reading these findings.

***Conclusions***

The investigation of the association of prenatal and perinatal factors with later onset of psychiatric conditions is particularly hard to perform from a methodological standpoint. This is even more true when such an evaluation is applied to quite rare conditions like AN(Hoek 2006). Notwithstanding, the available body of evidence supports maternal stress during pregnancy and preterm birth as associated with the development of mixed diagnoses of EDs; still, BN was consistently associated with maternal psychosocial stress during pregnancy. Finally, multiple factors were reported to have an impact on the onset of AN in the offspring: namely, higher maternal age, pre-eclampsia and eclampsia, multiparity, hypoxic complications, prematurity, or preterm birth (<32 weeks), and being small for gestational birth size. However, our review contributed also to shed light on the plethora of methodological inconsistencies and flaws that characterize these lines of research (e.g., inclusion biases, self-report assessments of the EDs, lack of a shared definition of prenatal/perinatal factors, to name a few), potentially generating new ideas on how to tackle these weaknesses and finally provide a meta-analysis on this clinically relevant topic. More studies on larger samples evaluating multiple factors with a longitudinal design – the most fruitful research methodology given the research questions - are necessary to draw more solid conclusions on this topic. Also, as reported earlier (Krug et al., 2013), a shared definition of risk factors is required; in this light, the classification system adopted in this review (i.e., maternal factors, pregnancy complications, obstetric complications, and neonatal factors) could be a starting point to promote the additional debate on prenatal and perinatal factors in the field of EDs in turn encouraging well-designed studies.

# **Declarations**

# **Funding**

No funding was associated with this study. All authors had access to all the data and all authors were responsible for the decision to submit the manuscript.

**Conflict of Interests**

Authors and co-authors have no conflict of interest.

# **Contributors**

E.M. and F.C. did the literature search, data extraction, scoring and writing. All the other authors equally took part in the subsequent review and revision of the manuscript.

**Availability of data and material**

Not applicable.

**Code availability**

Not applicable.

**Ethics Approval**

Not applicable.

**Consent to participate**

Not applicable.

**Consent for pubblication**

Not applicable.

# **Bibliography**

Abbott PW, Gumusoglu SB, Bittle J, et al (2018) Prenatal stress and genetic risk: How prenatal stress interacts with genetics to alter risk for psychiatric illness. Psychoneuroendocrinology 90:9–21. https://doi.org/10.1016/j.psyneuen.2018.01.019

Ali A, Cui X, Eyles D (2018) Developmental vitamin D deficiency and autism: Putative pathogenic mechanisms. J Steroid Biochem Mol Biol 175:108–118. https://doi.org/10.1016/j.jsbmb.2016.12.018

Allen KL, Byrne SM, Kusel MMH, et al (2013) Maternal vitamin D levels during pregnancy and offspring eating disorder risk in adolescence. Int J Eat Disord 46:669–676. https://doi.org/10.1002/eat.22147

Atiye M, Miettunen J, Raevuori‐Helkamaa A (2015) A Meta-Analysis of Temperament in Eating Disorders. Eur Eat Disord Rev 23:89–99. https://doi.org/10.1002/erv.2342

Baker JH, Lichtenstein P, Kendler KS (2009) Intrauterine testosterone exposure and risk for disordered eating. Br J Psychiatry J Ment Sci 194:375–376. https://doi.org/10.1192/bjp.bp.108.054692

Baker JH, Schaumberg K, Munn-Chernoff MA (2017) Genetics of Anorexia Nervosa. Curr Psychiatry Rep 19:84. https://doi.org/10.1007/s11920-017-0842-2

Berenbaum SA, Bryk KK, Nowak N, et al (2009) Fingers as a Marker of Prenatal Androgen Exposure. Endocrinology 150:5119–5124. https://doi.org/10.1210/en.2009-0774

Button E, Aldridge S (2007) Season of birth and eating disorders: patterns across diagnoses in a specialized eating disorders service. Int J Eat Disord 40:468–471

Cannon TD, Rosso IM, Hollister JM, et al (2000) A prospective cohort study of genetic and perinatal influences in the etiology of schizophrenia. Schizophr Bull 26:351–366

Chowdhury U, Gordon I, Lask B, et al (2003) Early-onset anorexia nervosa: is there evidence of limbic system imbalance? Int J Eat Disord 33:388–396. https://doi.org/10.1002/eat.10155

Clarke MC, Tanskanen A, Huttunen M, et al (2011) Increased risk of schizophrenia from additive interaction between infant motor developmental delay and obstetric complications: evidence from a population-based longitudinal study. Am J Psychiatry 168:1295–1302. https://doi.org/10.1176/appi.ajp.2011.11010011

Cloninger CR, Svrakic DM, Przybeck TR (1993) A psychobiological model of temperament and character. Arch Gen Psychiatry 50:975–990

Cnattingius S, Hultman CM, Dahl M, Sparén P (1999) Very preterm birth, birth trauma, and the risk of anorexia nervosa among girls. Arch Gen Psychiatry 56:634–638

Cohen-Bendahan CCC, Buitelaar JK, van Goozen SHM, et al (2005) Is there an effect of prenatal testosterone on aggression and other behavioral traits? A study comparing same-sex and opposite-sex twin girls. Horm Behav 47:230–237. https://doi.org/10.1016/j.yhbeh.2004.10.006

Cohen-Bendahan CCC, Buitelaar JK, van Goozen SHM, Cohen-Kettenis PT (2004) Prenatal exposure to testosterone and functional cerebral lateralization: a study in same-sex and opposite-sex twin girls. Psychoneuroendocrinology 29:911–916. https://doi.org/10.1016/j.psyneuen.2003.07.001

Connan F, Campbell IC, Katzman M, et al (2003) A neurodevelopmental model for anorexia nervosa. Physiol Behav 79:13–24

Coombs E, Brosnan M, Bryant-Waugh R, Skevington SM (2011) An investigation into the relationship between eating disorder psychopathology and autistic symptomatology in a non-clinical sample. Br J Clin Psychol 50:326–338. https://doi.org/10.1348/014466510X524408

Culbert KM, Breedlove SM, Burt SA, Klump KL (2008) Prenatal hormone exposure and risk for eating disorders: a comparison of opposite-sex and same-sex twins. Arch Gen Psychiatry 65:329–336. https://doi.org/10.1001/archgenpsychiatry.2007.47

Culbert KM, Breedlove SM, Sisk CL, et al (2013) The emergence of sex differences in risk for disordered eating attitudes during puberty: a role for prenatal testosterone exposure. J Abnorm Psychol 122:420–432. https://doi.org/10.1037/a0031791

Culbert KM, Breedlove SM, Sisk CL, et al (2015) Age differences in prenatal testosterone’s protective effects on disordered eating symptoms: developmental windows of expression? Behav Neurosci 129:18–36. https://doi.org/10.1037/bne0000034

Dalle Grave R (2011) Eating disorders: progress and challenges. Eur J Intern Med 22:153–160. https://doi.org/10.1016/j.ejim.2010.12.010

Eagles JM, Andrew JE, Johnston MI, et al (2001) Season of birth in females with anorexia nervosa in Northeast Scotland. Int J Eat Disord 30:167–175

Fairburn CG, Harrison PJ (2003) Eating disorders. Lancet Lond Engl 361:407–416. https://doi.org/10.1016/S0140-6736(03)12378-1

Favaro A, Tenconi E, Ceschin L, et al (2011) In utero exposure to virus infections and the risk of developing anorexia nervosa. Psychol Med 41:2193–2199. https://doi.org/10.1017/S0033291710002655

Favaro A, Tenconi E, Santonastaso P (2006) Perinatal factors and the risk of developing anorexia nervosa and bulimia nervosa. Arch Gen Psychiatry 63:82–88. https://doi.org/10.1001/archpsyc.63.1.82

Favaro A, Tenconi E, Santonastaso P (2008) The relationship between obstetric complications and temperament in eating disorders: a mediation hypothesis. Psychosom Med 70:372–377. https://doi.org/10.1097/PSY.0b013e318164604e

Feingold E, Sheir-Neiss G, Melnychuk J, et al (2002) Eating disorder symptomatology is not associated with pregnancy and perinatal complications in a cohort of adolescents who were born preterm. Int J Eat Disord 31:202–209

Foley DL, Thacker LR, Aggen SH, et al (2001) Pregnancy and perinatal complications associated with risks for common psychiatric disorders in a population-based sample of female twins. Am J Med Genet 105:426–431

Fumagalli M, Provenzi L, De Carli P, et al (2018) From early stress to 12-month development in very preterm infants: Preliminary findings on epigenetic mechanisms and brain growth. PloS One 13:e0190602. https://doi.org/10.1371/journal.pone.0190602

Galderisi S, Mucci A, Monteleone P, et al (2003) Neurocognitive functioning in subjects with eating disorders: the influence of neuroactive steroids. Biol Psychiatry 53:921–927

Gardener H, Spiegelman D, Buka SL (2009) Prenatal risk factors for autism: comprehensive meta-analysis. Br J Psychiatry J Ment Sci 195:7–14. https://doi.org/10.1192/bjp.bp.108.051672

Gaudio S, Olivo G, Zobel BB, Schiöth HB (2018) Altered cerebellar–insular–parietal–cingular subnetwork in adolescents in the earliest stages of anorexia nervosa: a network–based statistic analysis. Transl Psychiatry 8:1–10. https://doi.org/10.1038/s41398-018-0173-z

Geddes JR, Lawrie SM (1995) Obstetric complications and schizophrenia: a meta-analysis. Br J Psychiatry J Ment Sci 167:786–793

Geddes JR, Verdoux H, Takei N, et al (1999) Schizophrenia and complications of pregnancy and labor: an individual patient data meta-analysis. Schizophr Bull 25:413–423

Georgieff MK (2006) The effect of maternal diabetes during pregnancy on the neurodevelopment of offspring. Minn Med 89:44–47

Giannopoulou I, Pagida MA, Briana DD, Panayotacopoulou MT (2018) Perinatal hypoxia as a risk factor for psychopathology later in life: the role of dopamine and neurotrophins. Horm Athens Greece 17:25–32. https://doi.org/10.1007/s42000-018-0007-7

Gillberg C, Råstam M, Gillberg IC (1994) Anorexia nervosa: physical health and neurodevelopment at 16 and 21 years. Dev Med Child Neurol 36:567–575

Goodman A, Heshmati A, Malki N, Koupil I (2014) Associations between birth characteristics and eating disorders across the life course: findings from 2 million males and females born in Sweden, 1975-1998. Am J Epidemiol 179:852–863. https://doi.org/10.1093/aje/kwt445

Hoek HW (2006) Incidence, prevalence and mortality of anorexia nervosa and other eating disorders. Curr Opin Psychiatry 19:389–394. https://doi.org/10.1097/01.yco.0000228759.95237.78

Hong Q, Pluye P, Fàbregues S (2018) Mixed Methods Appraisal Tool (MMAT)

Jacobi C, Fittig E, Bryson SW, et al (2011) Who is really at risk? Identifying risk factors for subthreshold and full syndrome eating disorders in a high-risk sample. Psychol Med 41:1939–1949. https://doi.org/10.1017/S0033291710002631

Jones C, Pearce B, Barrera I, Mummert A (2017) Fetal programming and eating disorder risk. J Theor Biol 428:26–33. https://doi.org/10.1016/j.jtbi.2017.05.028

Kampman O, Poutanen O (2011) Can onset and recovery in depression be predicted by temperament? A systematic review and meta-analysis. J Affect Disord 135:20–27. https://doi.org/10.1016/j.jad.2010.12.021

Katzman DK, Zipursky RB, Lambe EK, Mikulis DJ (1997) A longitudinal magnetic resonance imaging study of brain changes in adolescents with anorexia nervosa. Arch Pediatr Adolesc Med 151:793–797

King JA, Frank GKW, Thompson PM, Ehrlich S (2018) Structural Neuroimaging of Anorexia Nervosa: Future Directions in the Quest for Mechanisms Underlying Dynamic Alterations. Biol Psychiatry 83:224–234. https://doi.org/10.1016/j.biopsych.2017.08.011

Klump KL, Gobrogge KL, Perkins PS, et al (2006) Preliminary evidence that gonadal hormones organize and activate disordered eating. Psychol Med 36:539–546. https://doi.org/10.1017/S0033291705006653

Krug I, Taborelli E, Sallis H, et al (2013) A systematic review of obstetric complications as risk factors for eating disorder and a meta-analysis of delivery method and prematurity. Physiol Behav 109:51–62. https://doi.org/10.1016/j.physbeh.2012.11.003

Lindberg L, Hjern A (2003) Risk factors for anorexia nervosa: a national cohort study. Int J Eat Disord 34:397–408. https://doi.org/10.1002/eat.10221

Lindström K, Lagerroos P, Gillberg C, Fernell E (2006) Teenage outcome after being born at term with moderate neonatal encephalopathy. Pediatr Neurol 35:268–274. https://doi.org/10.1016/j.pediatrneurol.2006.05.003

Lofrano-Prado MC, Prado WL do, Barros MVG de, et al (2015) Obstetric complications and mother’s age at delivery are predictors of eating disorder symptoms among Health Science college students. Einstein Sao Paulo Braz 13:525–529. https://doi.org/10.1590/S1679-45082015AO3366

Lydecker JA, Pisetsky EM, Mitchell KS, et al (2012) Association between co-twin sex and eating disorders in opposite sex twin pairs: evaluations in North American, Norwegian, and Swedish samples. J Psychosom Res 72:73–77. https://doi.org/10.1016/j.jpsychores.2011.05.014

Matinolli H-M, Männistö S, Sipola-Leppänen M, et al (2016) Body image and eating behavior in young adults born preterm. Int J Eat Disord 49:572–580. https://doi.org/10.1002/eat.22553

McGrath JJ, Eyles DW, Pedersen CB, et al (2010) Neonatal vitamin D status and risk of schizophrenia: a population-based case-control study. Arch Gen Psychiatry 67:889–894. https://doi.org/10.1001/archgenpsychiatry.2010.110

McGrath JJ, Hannan AJ, Gibson G (2011) Decanalization, brain development and risk of schizophrenia. Transl Psychiatry 1:e14. https://doi.org/10.1038/tp.2011.16

Meyer JS, Hamel AF (2014) Models of stress in nonhuman primates and their relevance for human psychopathology and endocrine dysfunction. ILAR J 55:347–360. https://doi.org/10.1093/ilar/ilu023

Micali N, Kothari R, Nam KW, et al (2015) Eating disorder psychopathology, brain structure, neuropsychological correlates and risk mechanisms in very preterm young adults. Eur Eat Disord Rev J Eat Disord Assoc 23:147–155. https://doi.org/10.1002/erv.2346

Moher D, Liberati A, Tetzlaff J, et al (2009) Preferred reporting items for systematic reviews and meta-analyses: the PRISMA statement. PLoS Med 6:e1000097. https://doi.org/10.1371/journal.pmed.1000097

Montgomery SM, Ehlin A, Ekbom A (2005) Smoking during pregnancy and bulimia nervosa in offspring. J Perinat Med 33:206–211. https://doi.org/10.1515/JPM.2005.038

Morgan JF, Lacey JH (2000) Season of birth and bulimia nervosa. Int J Eat Disord 27:452–458. https://doi.org/10.1002/(SICI)1098-108X(200005)27:4<452::AID-EAT10>3.0.CO;2-M

Nicholls DE, Viner RM (2009) Childhood risk factors for lifetime anorexia nervosa by age 30 years in a national birth cohort. J Am Acad Child Adolesc Psychiatry 48:791–799. https://doi.org/10.1097/CHI.0b013e3181ab8b75

Nist MD, Harrison TM, Steward DK (2019) The biological embedding of neonatal stress exposure: A conceptual model describing the mechanisms of stress-induced neurodevelopmental impairment in preterm infants. Res Nurs Health 42:61–71. https://doi.org/10.1002/nur.21923

Nosarti C, Reichenberg A, Murray RM, et al (2012) Preterm birth and psychiatric disorders in young adult life. Arch Gen Psychiatry 69:E1-8. https://doi.org/10.1001/archgenpsychiatry.2011.1374

Pace R, Pluye P, Bartlett G, et al (2012) Testing the reliability and efficiency of the pilot Mixed Methods Appraisal Tool (MMAT) for systematic mixed studies review. Int J Nurs Stud 49:47–53. https://doi.org/10.1016/j.ijnurstu.2011.07.002

Pagnin D, Zamboni Grecco ML, Furtado EF (2019) Prenatal alcohol use as a risk for attention-deficit/hyperactivity disorder. Eur Arch Psychiatry Clin Neurosci 269:681–687. https://doi.org/10.1007/s00406-018-0946-7

Pluye P, Gagnon M-P, Griffiths F, Johnson-Lafleur J (2009) A scoring system for appraising mixed methods research, and concomitantly appraising qualitative, quantitative and mixed methods primary studies in Mixed Studies Reviews. Int J Nurs Stud 46:529–546. https://doi.org/10.1016/j.ijnurstu.2009.01.009

Quinton SJ, Smith AR, Joiner T (2011) The 2 to 4 digit ratio (2D:4D) and eating disorder diagnosis in women. Personal Individ Differ 51:402–405. https://doi.org/10.1016/j.paid.2010.07.024

Raevuori A, Kaprio J, Hoek HW, et al (2008) Anorexia and bulimia nervosa in same-sex and opposite-sex twins: lack of association with twin type in a nationwide study of Finnish twins. Am J Psychiatry 165:1604–1610. https://doi.org/10.1176/appi.ajp.2008.08030362

Raevuori A, Linna MS, Keski-Rahkonen A (2014) Prenatal and perinatal factors in eating disorders: a descriptive review. Int J Eat Disord 47:676–685. https://doi.org/10.1002/eat.22323

Rapoport JL, Giedd JN, Gogtay N (2012) Neurodevelopmental model of schizophrenia: update 2012. Mol Psychiatry 17:1228–1238. https://doi.org/10.1038/mp.2012.23

Razaz N, Cnattingius S (2018) Association between maternal body mass index in early pregnancy and anorexia nervosa in daughters. Int J Eat Disord 51:906–913. https://doi.org/10.1002/eat.22921

Resnick SM, Gottesman II, McGue M (1993) Sensation seeking in opposite-sex twins: an effect of prenatal hormones? Behav Genet 23:323–329

Rivera HM, Christiansen KJ, Sullivan EL (2015) The role of maternal obesity in the risk of neuropsychiatric disorders. Front Neurosci 9:194. https://doi.org/10.3389/fnins.2015.00194

Romero-Martínez A, Moya-Albiol L (2014) Prenatal testosterone of progenitors could be involved in the etiology of both anorexia nervosa and autism spectrum disorders of their offspring. Am J Hum Biol Off J Hum Biol Counc 26:863–866. https://doi.org/10.1002/ajhb.22597

Sacks KN, Friger M, Shoham-Vardi I, et al (2016) Prenatal exposure to gestational diabetes mellitus as an independent risk factor for long-term neuropsychiatric morbidity of the offspring. Am J Obstet Gynecol 215:380.e1–7. https://doi.org/10.1016/j.ajog.2016.03.030

Shoebridge P, Gowers SG (2000) Parental high concern and adolescent-onset anorexia nervosa. A case-control study to investigate direction of causality. Br J Psychiatry J Ment Sci 176:132–137. https://doi.org/10.1192/bjp.176.2.132

Smith AR, Hawkeswood SE, Joiner TE (2010) The measure of a man: associations between digit ratio and disordered eating in males. Int J Eat Disord 43:543–548. https://doi.org/10.1002/eat.20736

St-Hilaire A, Steiger H, Liu A, et al (2015) A prospective study of effects of prenatal maternal stress on later eating-disorder manifestations in affected offspring: preliminary indications based on the Project Ice Storm cohort. Int J Eat Disord 48:512–516. https://doi.org/10.1002/eat.22391

Su X, Liang H, Yuan W, et al (2016) Prenatal and early life stress and risk of eating disorders in adolescent girls and young women. Eur Child Adolesc Psychiatry 25:1245–1253. https://doi.org/10.1007/s00787-016-0848-z

Taborelli E, Krug I, Karwautz A, et al (2013) Maternal Anxiety, Overprotection and Anxious Personality as Risk Factors for Eating Disorder: A Sister Pair Study. Cogn Ther Res 37:820–828. https://doi.org/10.1007/s10608-012-9518-8

Tenconi E, Santonastaso P, Monaco F, Favaro A (2015) Obstetric complications and eating disorders: a replication study. Int J Eat Disord 48:424–430. https://doi.org/10.1002/eat.22304

Trace SE, Baker JH, Peñas-Lledó E, Bulik CM (2013) The genetics of eating disorders. Annu Rev Clin Psychol 9:589–620. https://doi.org/10.1146/annurev-clinpsy-050212-185546

Vellisca MY, Latorre JI, Santed MA, Reales JM (2013) Absence of seasonal pattern of birth in patients with anorexia nervosa. Int J Eat Disord 46:86–88. https://doi.org/10.1002/eat.22046

Verdoux H, Geddes JR, Takei N, et al (1997) Obstetric complications and age at onset in schizophrenia: an international collaborative meta-analysis of individual patient data. Am J Psychiatry 154:1220–1227. https://doi.org/10.1176/ajp.154.9.1220

Waller G, Watkins B, Potterton C, et al (2002) Pattern of birth in adults with anorexia nervosa. J Nerv Ment Dis 190:752–756. https://doi.org/10.1097/01.NMD.0000038170.13117.D5

Watkins B, Willoughby K, Waller G, et al (2002) Pattern of birth in anorexia nervosa. I: Early-onset cases in the United Kingdom. Int J Eat Disord 32:11–17. https://doi.org/10.1002/eat.10057

Wehkalampi K, Hovi P, Strang-Karlsson S, et al (2010) Reduced body size and shape-related symptoms in young adults born preterm with very low birth weight: Helsinki study of very low birth weight adults. J Pediatr 157:421–427, 427.e1. https://doi.org/10.1016/j.jpeds.2010.02.045

Whittemore R, Knafl K (2005) The integrative review: updated methodology. J Adv Nurs 52:546–553. https://doi.org/10.1111/j.1365-2648.2005.03621.x

Winje E, Torgalsbøen A-K, Brunborg C, Lask B (2013) Season of birth bias and anorexia nervosa: results from an international collaboration. Int J Eat Disord 46:340–345. https://doi.org/10.1002/eat.22060

**Supplementary Material**

**Supplementary Table 1 – Characteristics of the studies included in the systematic review.**

|  |  |  |  |  | **MMAT v.2018 Quality Rating**  ◆ = Yes ◇= No ◈= Can’t Tell | | | | |
| --- | --- | --- | --- | --- | --- | --- | --- | --- | --- |
| **Authors** | **Title** | **Year** | **Study Type** | **Psychiatric Assessment** | **Sample Selection** | **Data Collection** | **Outcome Data Completeness** | **Confounder Analysis** | **Change in Exposure Status** |
|  |  |  |  |  |  |  |  |  |  |
| Cnattingius et al. | Very preterm birth, birth trauma, and the risk of anorexia nervosa among girls | 1999 | Case-Control Study with random selected control (Swedish Impatient Register – 1973-1984) | ICD-9 | ◆ | ◆ | ◆ | ◆ | ◆ |
| Morgan et al. | Season of birth and bulimia nervosa | 2000 | Case-Control Study | DSM-IV criteria | ◆ | ◆ | ◆ | ◇ | ◆ |
| Shoebridge & Gowers et al. | Parental high concern and adolescent-onset of anorexia nervosa. A case-control study to investigate direction of causality | 2000 | Case-control study | DSM-III-R | ◈ | ◆ | ◇ | ◇ | ◆ |
| Foley et al. | Pregnancy and  perinatal complications associated with risks for common psychiatric disorders  in a population-based sample of female twins | 2001 | Twin Cohort Study (Virginia Twin Registry, n=2352) | Simplified Structured Clinial Interview DSM-III-R for AN and BN and clinical diagnosis of AN/BN or AN/BN phenotypes for bdAN and bdBN | ◈ | ◆ | ◆ | ◇ | ◆ |
| Feingold et al. | Eating disorder symptomatology is not associated with pregnancy and perinatal complications in a cohort of adolescents who were born preterm | 2002 | HIstorical Prospective Cohort (Thomas Jefferson University Hospital 1979-1981, n=86) | Self reported EDI-2, EAT and other psychiatric scales | ◇ | ◇ | ◆ | ◇ | ◆ |
|  |  |  |  |  |  |  |  |  |  |
| Lindberg et al. | Risk factors for anorexia nervosa: A national cohort  study | 2003 | Population Cohort Study (Swedish Register Data 1973-1982 n=989,871) | ICD-9 and ICD-10 | ◆ | ◆ | ◆ | ◈ | ◆ |
| Montgomery et al. | Smoking during pregnancy and bulimia  nervosa in offspring | 2005 | Cohort Study (British Cohort Study 1970 n=4046) | Self report of previous diagnosis | ◆ | ◇ | ◆ | ◇ | ◆ |
| Favaro et al. | Perinatal factors and the risk of developing anorexia and bulimia nervosa | 2006 | Birth Cohort Study, (Padua Birth Cohort 1971-1979) | DSM-IV criteria | ◆ | ◆ | ◆ | ◆ | ◆ |
| Klump et al. | Preliminary  evidence that gonadal hormones organize and activate disordered  eating | 2006 | Twin Cohort Study (Michigan State Twin Study) | MEBS | ◈ | ◇ | ◆ | ◆ | ◇ |
| Culbert et al. | Prenatal hormone exposure  and risk for eating disorders: A comparison of opposite-sex and same-sex  twins | 2008 | Twin Cohort Study (Michigan State Twin Study) | MEBS | ◈ | ◇ | ◆ | ◇ | ◆ |
| Favaro et al. | The relationship between obstetric complications and temperament in eating disorders: a mediation hypothesis | 2008 | Birth Cohort Study, (Padua Birth Cohort 1971-1979) | DSM-IV criteria | ◆ | ◆ | ◆ | ◆ | ◈ |
| Raevuori et al. | Anorexia and bulimia nervosa in same-sex and opposite-sex twins: lack of  association with twin type in a nationwide study of Finnish twins | 2008 | Twin Cohort Study (FinnTwin 16, 1975-1979 sample n=2426) | EDI screening and then DSM-IV criteria | ◆ | ◆ | ◆ | ◇ | ◆ |
| Baker et al. | Intrauterine testosterone exposure and  risk for disordered eating | 2009 | Swedish Twin Study of Child and Adolescent Development (TCHAD) 1985-1986 | EDI-2 | ◆ | ◇ | ◆ | ◇ | ◆ |
| Nicholls et al. | Childhood risk factors for lifetime anorexia nervosa by age 30 years in a national birth cohort | 2009 | Prospective Birth Cohort (British Cohort Study, n=16,567) | Self report of past diagnosis | ◆ | ◇ | ◆ | ◇ | ◆ |
| Smith et al. | The measure of a man: Associations  between digit ratio and disordered eating in males | 2010 | Cross sectional observational Study | MBAS, EDEQ-4 | ◇ | ◇ | ◆ | ◇ | ◆ |
| Wehkalampi et al. | Reduced body size and shape-related symptoms in young  adults born preterm with very low birth weight: Helsinki study of very low  birth weight adults | 2010 | Case-Control Study (Helsinki Study of Very Low Birth Weight Adults 1978-1985 (n=255) | EDI-2 | ◆ | ◇ | ◆ | ◇ | ◆ |
| Coombs et al. | An investigation into the relationship between eating disorder psychopathology and autistic symptomatology in a non-clinical sample | 2011 | Observational study on a Midlands High School (UK) students | EAT-26 | ◇ | ◇ | ◆ | ◇ | ◆ |
| Favaro et al. | In utero  exposure to virus infections and the risk of developing anorexia nervosa | 2011 | Birth Cohort Study, (Padua Birth Cohort 1970-1984, n=27,682) | DSM-IV criteria | ◆ | ◆ | ◆ | ◇ | ◆ |
| Quinton et al. | The 2 to 4 digit ratio (2D:4D) and eating disorder  diagnosis in women | 2011 | Case-Control Study | Eating Disorder Association database with self report of ED subtype (ANr, ANbp, BN) | ◈ | ◇ | ◆ | ◇ | ◆ |
| Lydecker et al. | Association between co-twin sex and eating disorders in  opposite sex twin pairs: Evaluations in North American, Norwegian, and  Swedish samples | 2012 | Three twin Cohort Study (MATR (US), NIPHTP (Norway), STAGE (Sweden)) | DSM-IV criteria adapted to self-questionnaires | ◆ | ◇ | ◆ | ◆ | ◆ |
| Nosarti et al. | Preterm birth and psychiatric disorders in young adult life | 2012 | Historical population-based cohort study (Swedish Birth Register 1973-1985 and Hospital Discharge Register n= 1,301,522) | ICD-8, ICD-9, ICD-10 | ◆ | ◇ | ◆ | ◆ | ◆ |
|  |  |  |  |  |  |  |  |  |  |
| Allen et al. | Maternal vitamin D  levels during pregnancy and offspring eating disorder risk in adolescence | 2013 | Population Cohort (Western Australian Pregnancy Cohort (Raine) n=2900) | Child Eating Disorder Examination and EDE-Q adapted with DSM-IV and 5 criteria | ◆ | ◇ | ◆ | ◇ | ◆ |
| Culbert et al. | The emergence of sex differences in risk for disordered eating attitudes during puberty: a role for prenatal testosterone exposure | 2013 | Twin Cohort Study (Michigan State Twin Study) | MEBS, EDE-Q | ◈ | ◇ | ◆ | ◇ | ◆ |
|  |  |  |  |  |  |  |  |  |  |
| Taborelli et al. | Maternal Anxiety, Overprotection and Anxious Personality  as Risk Factors for Eating Disorder: A Sister Pair Study | 2013 | Case-Control Study (Sister Pair Study) | DSM-IV criteria | ◆ | ◆ | ◆ | ◇ | ◆ |
| Vellisca et al. | Absence of seasonal pattern of birth in patients with anorexia nervosa | 2013 | Case-Control Study | DSM-IV criteria | ◆ | ◆ | ◆ | ◇ | ◆ |
| Winje et al. | Season of birth bias and anorexia  nervosa: Results from an international collaboration | 2013 | Case-Control Study | DSM-IV, ICD-10, Great Ormond Street Criteria | ◆ | ◆ | ◆ | ◇ | ◆ |
| Goodman et al. | Associations between birth characteristics  and eating disorders across the life course: Findings from 2 million  males and females born in Sweden | 2014 | Population Cohort Study (Swedish Register Data, 1975-1998, n=2,135,279) | ICD-9 and ICD-10 | ◆ | ◆ | ◆ | ◆ | ◆ |
| Romero-Martínez et al.. | Prenatal testosterone of progenitors could be involved in the etiology of both anorexia nervosa and autism spectrum disorders of their offspring | 2014 | Observational Study | DSM-IV-R | ◆ | ◇ | ◈ | ◆ | ◇ |
| Culbert et al. | Age differences in prenatal testosterone's protective effects on disordered eating symptoms: developmental windows of expression? | 2015 | Michigan State Univeristy Twin Registry | MEBS, EDE-Q | ◆ | ◇ | ◆ | ◈ | ◆ |
| Lofrano-Prado et al | Obstetric complications and mother’s age at delivery  are predictors of eating disorder symptoms among  Health Science college students | 2015 | Cross-sectional observational study | EAT-26, BITE | ◆ | ◇ | ◆ | ◆ | ◆ |
| Micali et al. | Eating Disorder Psychopathology, Brain Structure,  Neuropsychological Correlates and Risk Mechanisms in Very  Preterm Young Adults | 2015 | Cohort Study (Very Preterm Cohort n=476) | EDE-Q | ◆ | ◇ | ◇ | ◇ | ◆ |
| St-Hilaire et al. | A Prospective Study of Effects of Prenatal Maternal Stress  on Later Eating-Disorder Manifestations in Affected  Offspring: Preliminary Indications Based on the Project  Ice Storm Cohort | 2015 | Cohort Study (Project Ice Storm Cohort, n=54) | EAT-26 | ◇ | ◇ | ◆ | ◆ | ◆ |
| Tenconi et al. | Obstetric complications and eating disorders: a replication study | 2015 | Birth Cohort Study, (Padua new Birth Cohort 1969-1997 and previous 1971-1979) | DSM-IV criteria | ◆ | ◆ | ◆ | ◆ | ◆ |
| Mattinolli et al. | Body Image and Eating Behavior in Young Adults  Born Preterm | 2016 | Population Cohort Study (ESTER 1985-1989 e AYLS 1985-1986) | EDI-2 | ◆ | ◇ | ◆ | ◆ | ◆ |
| Sacks et al. | Prenatal exposure to gestational diabetes mellitus as an  independent risk factor for long-term neuropsychiatric  morbidity of the offspring | 2016 | Population Cohort Study (Soroka University Medical Center, Israel 1991-2014 (n=231,271) | ICD-9 | ◆ | ◇ | ◇ | ◇ | ◆ |
| Su et al. | Prenatal and early life stress and risk of eating disorders in adolescent girls and young women | 2016 | Population based cohort (Denmark 1973-2000 (n=1,034,539) and Sweden 1970-1997 (n=1,246,560)) | ICD-8, ICD-9, ICD-10 | ◆ | ◆ | ◆ | ◇ | ◆ |
| Razaz et al. | Association between maternal body mass index in early  pregnancy and anorexia nervosa in daughters | 2018 | Retrospective Swedish cohort study (1992-2002, n= 486,688) | ICD-9, ICD-10 | ◆ | ◆ | ◆ | ◆ | ◆ |

**MMAT v2018:** SS: sample selection, DC: data collection, ODC: outcome data completeness, CA: confounder analysis, CES: change in exposure status.

*AN* anorexia nervosa, *BN* bulimia nervosa, *HC* healthy controls, *ED* eating disorder, *EDI-2* Eating Disorder Inventory, *EAT-26* Eating Attitude Test, *BITE* Bulimic Investigatory Test, *MBAS* Male Body Attitude Test, *EDEQ-4* Eating Disorder Examination Questionnaire, *MEBS* Minnesota Eating Behaviors Survey, *FSS* female same sax twins, *FOS* female opposite sex twins, *MOS* male opposite sex twins, *MSS* male same sec twins, *SS* same sex, *OS* opposite sex, *CNS* central nervous system, *VPT* very preterm, *BMI* body mass index.
